# Supplementary figures and images for: Posttranslational insertion of small membrane proteins by the bacterial signal recognition particle
Source: PLoS Biol. 2020 Sep 30;18(9):e3000874. doi: 10.1371/journal.pbio.3000874 (PMC7549839; doi:10.1371/journal.pbio.3000874)

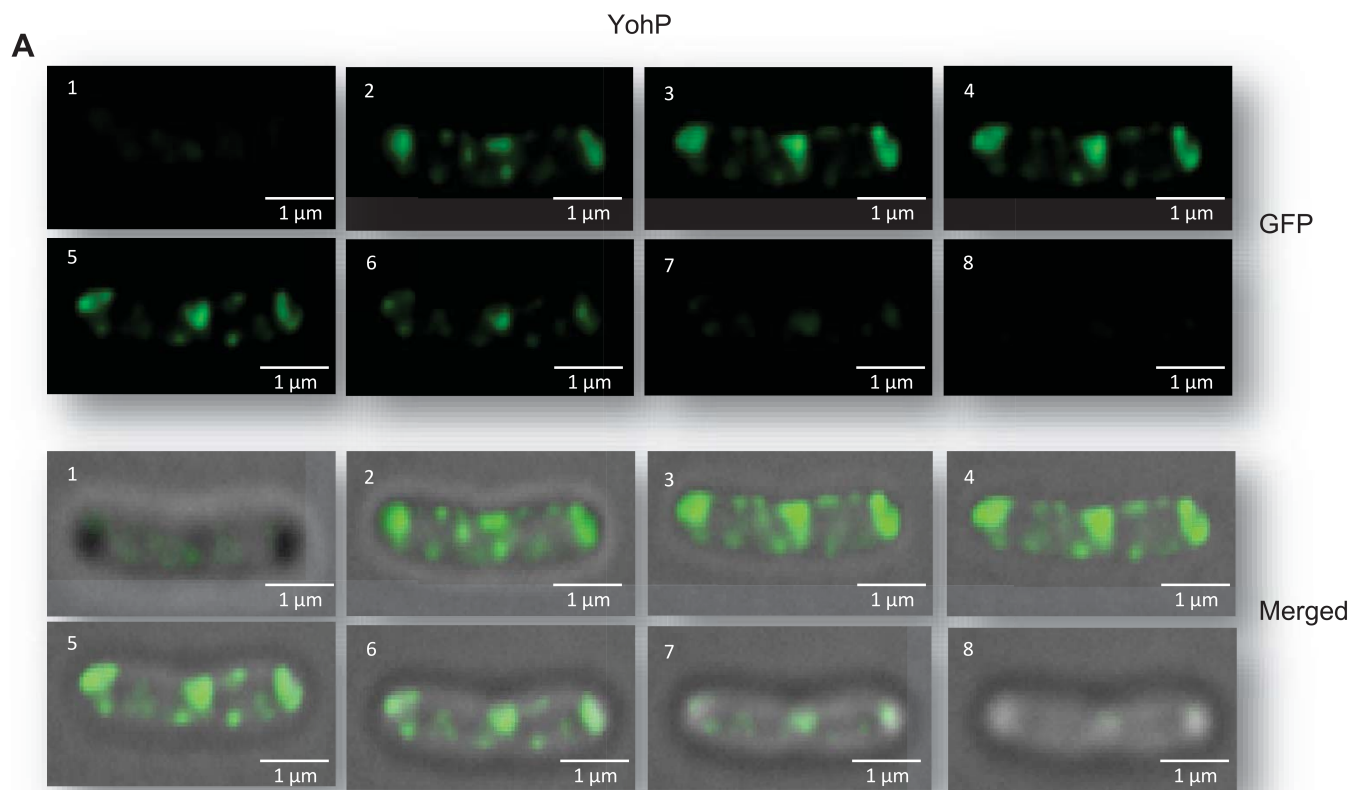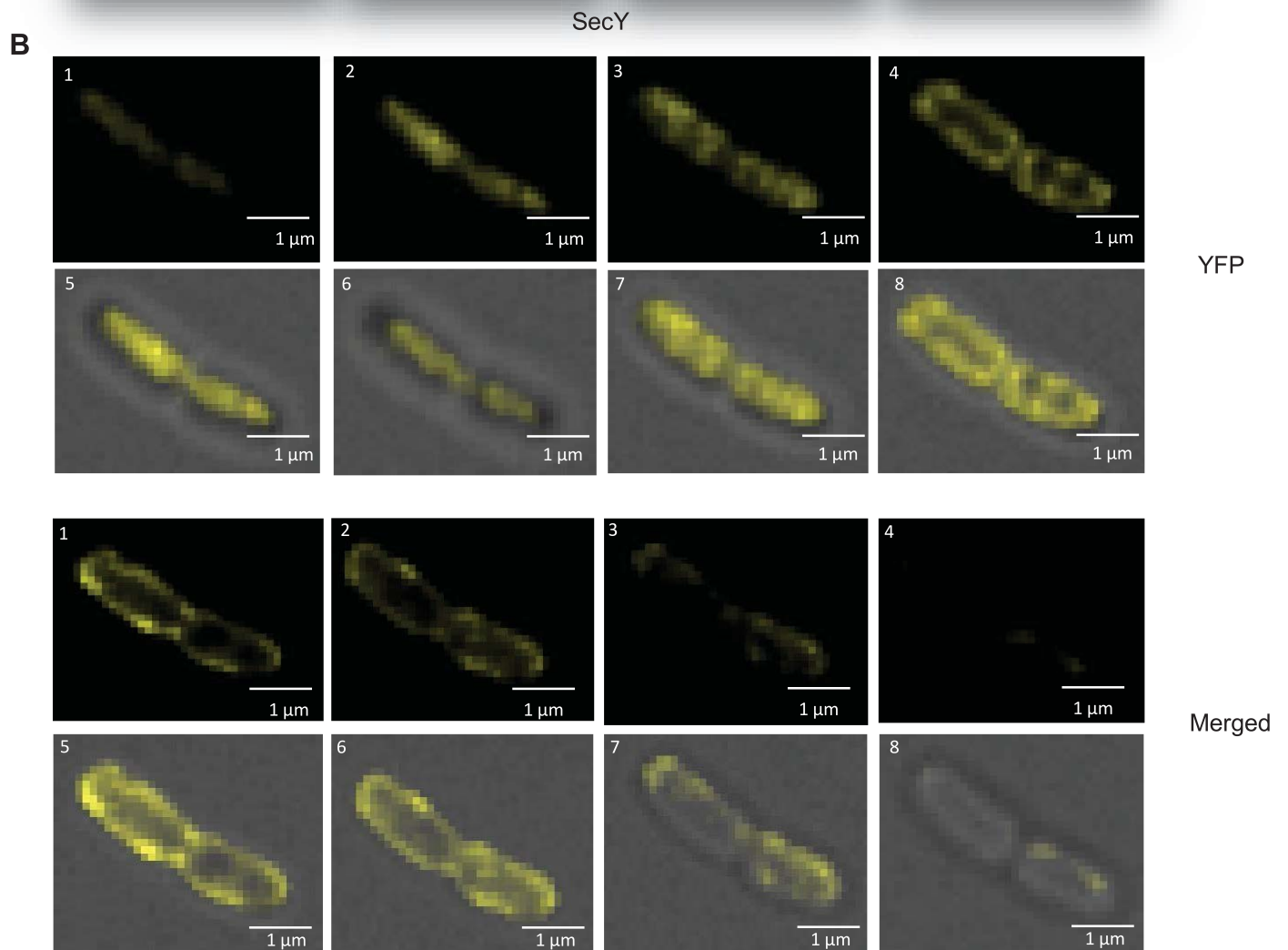

Supplement: S1 Fig — YohP-GFP and SecY-YFP were in vivo expressed, and imaging was performed with a DeltaVision Ultra High Resolution Widefield Microscope (GE Healthcare, Munich, Germany) at 100× magnification. Recording, using camera sCMOS pro edge (PCO, Kelheim, Germany), was performed using a 3-μm Z-scan with 0.1-μm sectioning, and the different scans of both the fluorescence channel and the merged fluorescence/bright-field picture are shown. GFP, green fluorescent protein; YFP, yellow fluorescent protein. (PDF) [file pbio.3000874.s001.pdf]

YchF

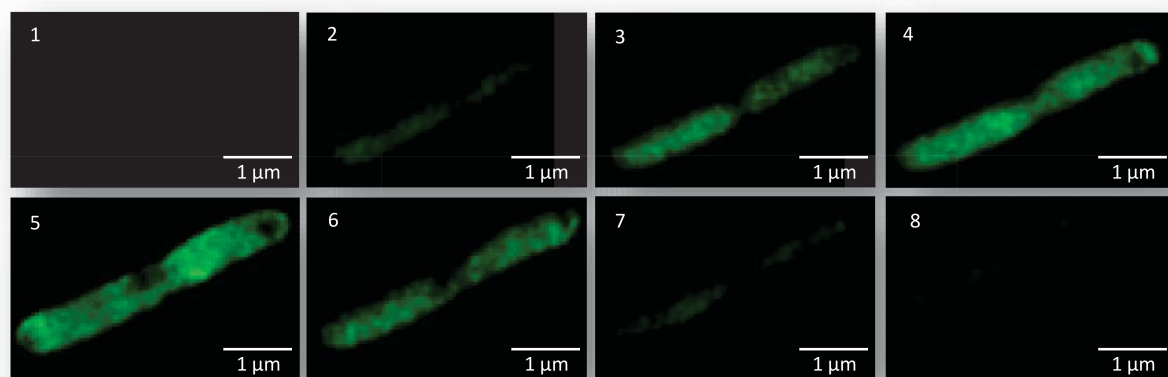

GFP

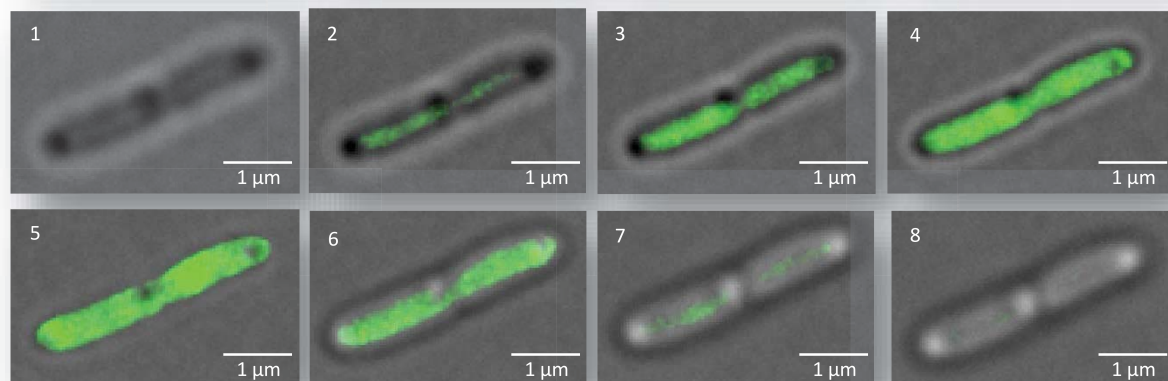

Merged

Supplement: S2 Fig — YchF-GFP was in vivo expressed and imaging was performed with a DeltaVision Ultra High Resolution Widefield Microscope (GE Healthcare, Munich, Germany) at 100× magnification. Recording, using camera sCMOS pro edge (PCO, Kelheim, Germany), was performed using a 3-μm Z-scan with 0.1-μm sectioning, and the different scans of both the fluorescence channel and the merged fluorescence/bright-field picture are shown. GFP, green fluorescent protein. (PDF) [file pbio.3000874.s002.pdf]

| fraction | whole cells |   | S30 | P30 | S150 | INV | OM |
|----------|-------------|---|-----|-----|------|-----|----|
| Ara      | -           | + |     |     |      |     |    |

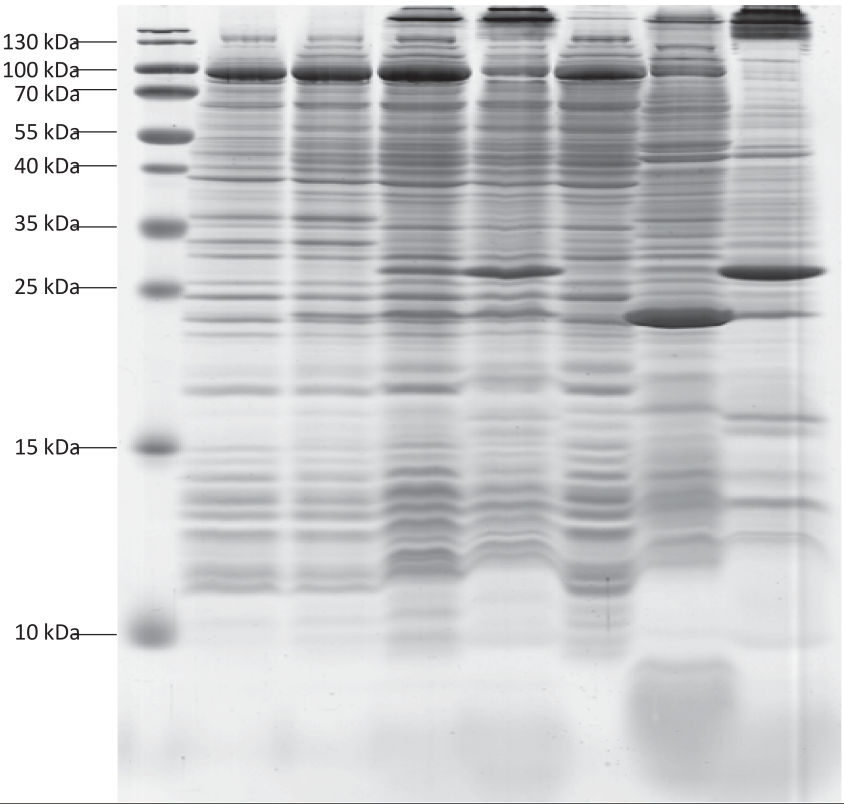

Supplement: S3 Fig — E. coli cells carrying pBad-YohPHis were induced with 0.2% arabinose when indicated and subsequently fractionated by differential centrifugation as described in the legend to Fig 1D. Of each fraction, an aliquot corresponding to 40 μg protein was separated by SDS-PAGE and stained with Coomassie blue. YohPHis, His-tagged YohP. (PDF) [file pbio.3000874.s003.pdf]

**A**

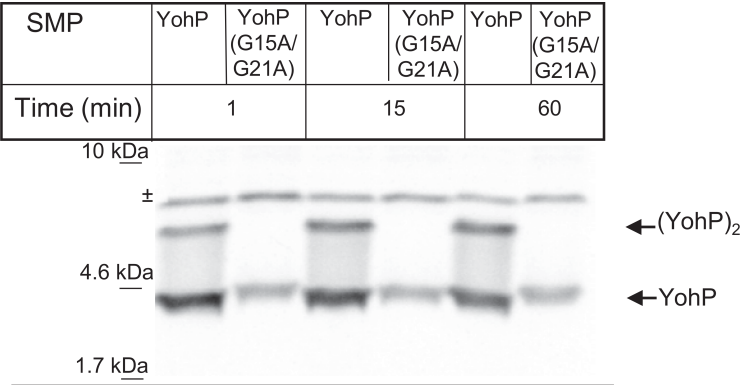

**B**

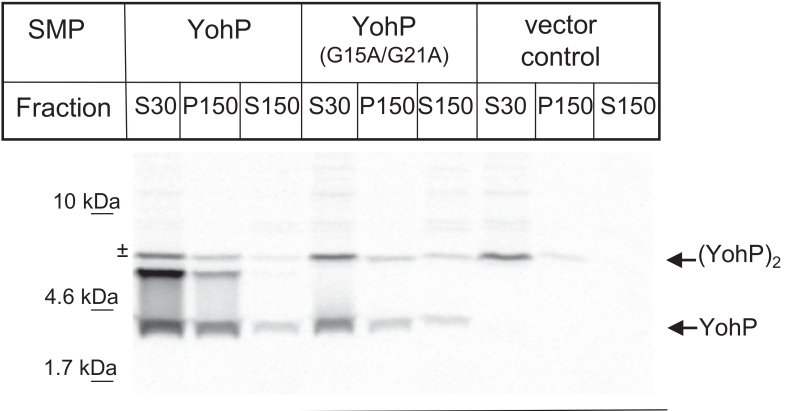

**C**

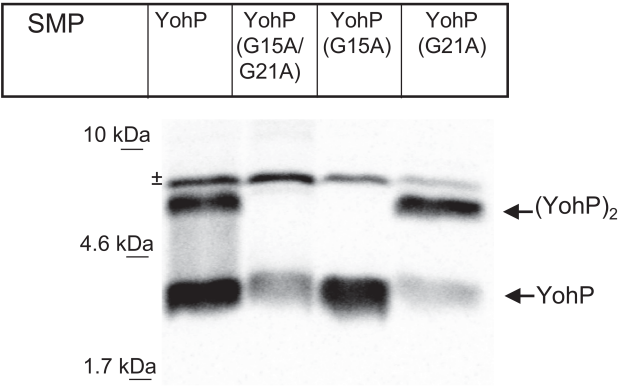

Supplement: S4 Fig — (A) YohP or the YohP(G15A/G21A) mutant were in vivo expressed and pulse-labeled. Whole cells were then TCA precipitated after the indicated time points, separated by SDS-PAGE and analyzed by autoradiography. ± refers to a nonspecifically labeled band. (B) As in (A), but cells were fractionated after cell breakage into an S30 (cytosol and membrane), S150 (cytosol), and P150 (membrane) fraction, as described in the legend to Fig 1. (C) YohP, the YohP(G15A/G21A) mutant, or the two single mutants YohP(G15A) and YohP(G21A) were in vivo expressed, pulse-labeled, and analyzed by autoradiography as above. TCA, trichloroacetic acid. (PDF) [file pbio.3000874.s004.pdf]

**A**

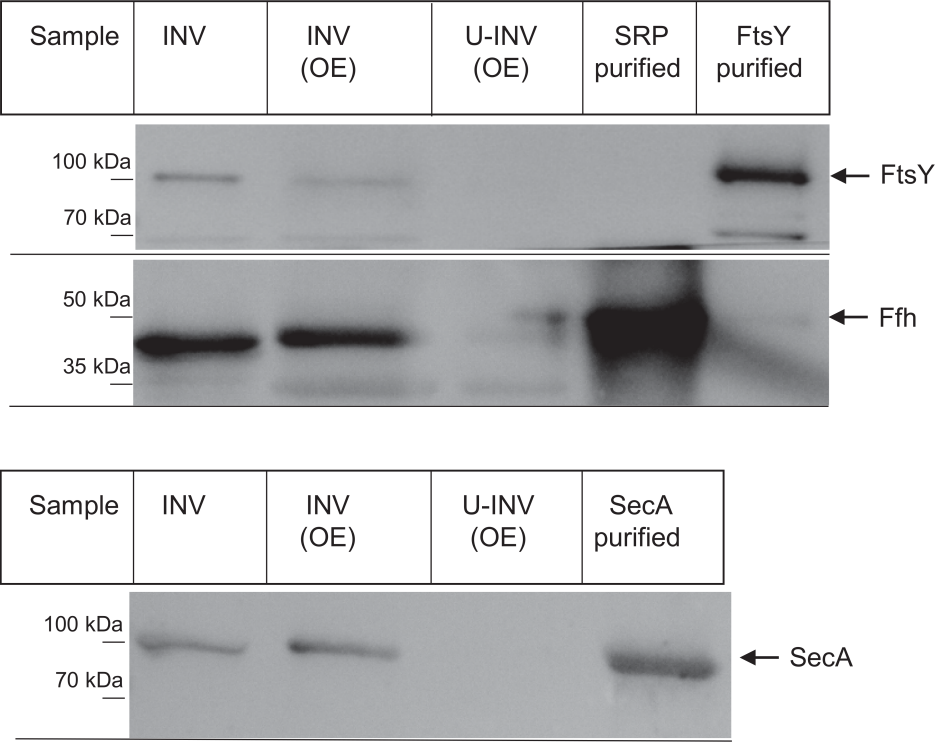

**B**

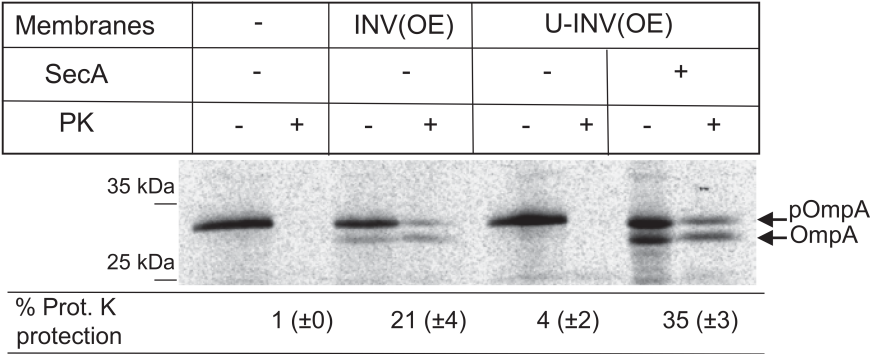

**C**

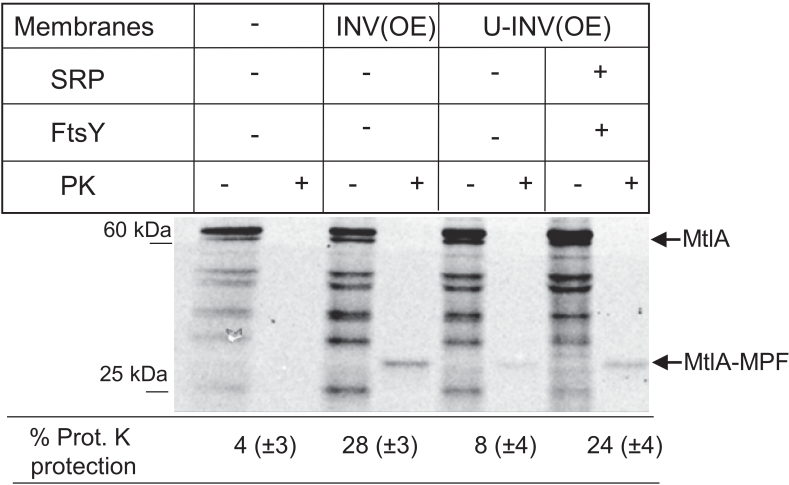

Supplement: S5 Fig — (A) Urea-treated vesicles were decorated after SDS-PAGE and western blotting with antibodies FtsY, Ffh (upper panel), or SecA (lower panel). (B) The translocation of OmpA into U-INV requires the presence of SecA. Translocation of OmpA was analyzed as described in Fig 3. (C) The integration of MtlA into U-INV requires the presence of FtsY and SRP. MtlA integration was analyzed as described in Fig 3. Underlying data for this figure can be found in S1 Data. INV, inner membrane vesicle; MtlA, mannitol permease; OE, INV from an SecYEG-overexpressing strain; SRP, signal recognition particle; U-INV, urea-treated INV. (PDF) [file pbio.3000874.s005.pdf]

**A**

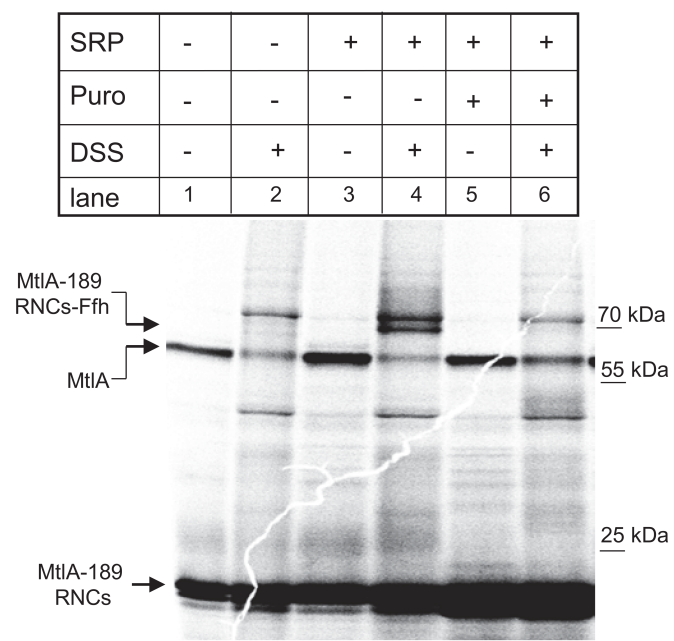

**B**

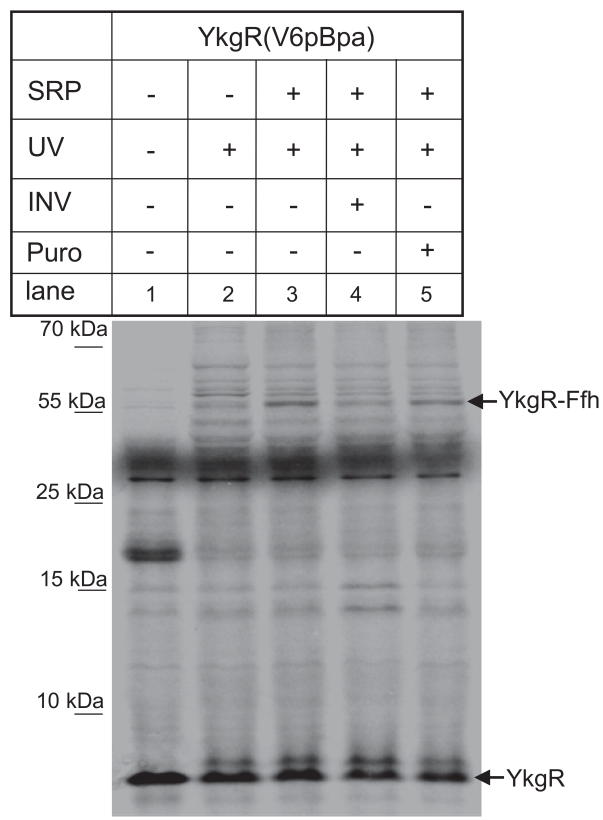

Supplement: S6 Fig — (A) MtlA-189 RNCs (189 amino acids) of the inner membrane protein MtlA were in vitro synthesized using an antisense oligonucleotide approach [1]. When indicated, purified SRP was added to MtlA-RNCs. Cross-linking was induced by the addition of DSS, an amine-reactive cross-linker that had been used before for monitoring MtlA-SRP contacts [1, 2]. When indicated, puromycin (1 mM) was added prior to DSS cross-linking. Note that full-length MtlA is also visible, because the antisense oligonucleotide approach for generating RNCs does not allow for a complete suppression of full-length protein synthesis. The identity of the Ffh-MtlA-189 RNC cross-link has been validated in multiple studies [1–3]. (B) YkgR(V6pBpa)His was in vitro synthesized and, when indicated, incubated with purified SRP and UV-exposed in the presence of INV or after puromycin treatment. Samples in (A) and (B) were subsequently TCA precipitated, separated by SDS-PAGE, and visualized by autoradiography. At least two biological replicates were performed, and a representative gel is shown. DSS, disuccinimidyl suberate; MtlA, mannitol permease; RNC, ribosome-associated nascent chain; SRP, signal recognition particle; TCA, trichloroacetic acid; YkgR(V6pBpa)His, YkgR containing the UV-reactive cross-linker para-benzoyl-L-phenylalanine at position 6. (PDF) [file pbio.3000874.s006.pdf]

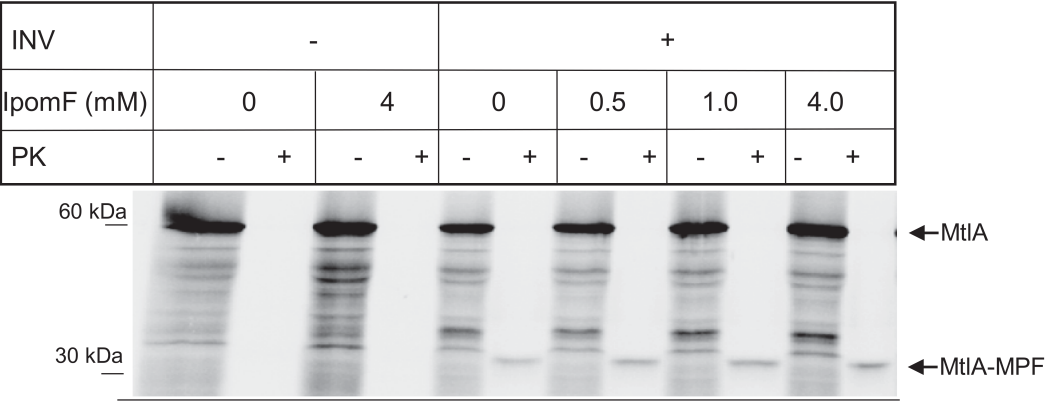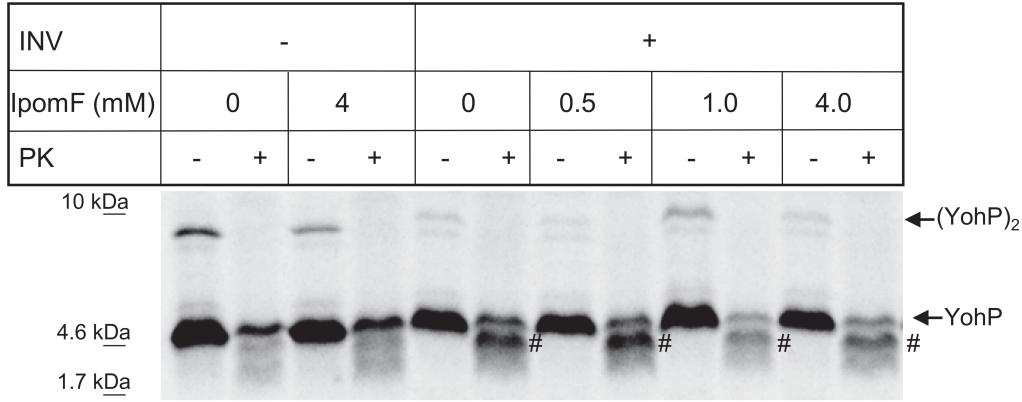

Supplement: S7 Fig — MtlA and YohP were in vitro synthesized, and membrane insertion was analyzed into INVs that were pretreated with different concentrations of the inhibitor IpomF or with DMSO as control (0 mM IpomF). Indicated is the MtlA-MPF, which results from degradation of the approximately 30-kDa cytoplasmic domain by proteinase K and the membrane-protected fragment of YohP (#). Quantification of several independent experiments is shown in Fig 6B. INV, inner membrane vesicle; IpomF, Ipomoeassin F; MtlA, mannitol permease; MtlA-MPF, membrane-protected fragment of MtlA. (PDF) [file pbio.3000874.s007.pdf]

**A**

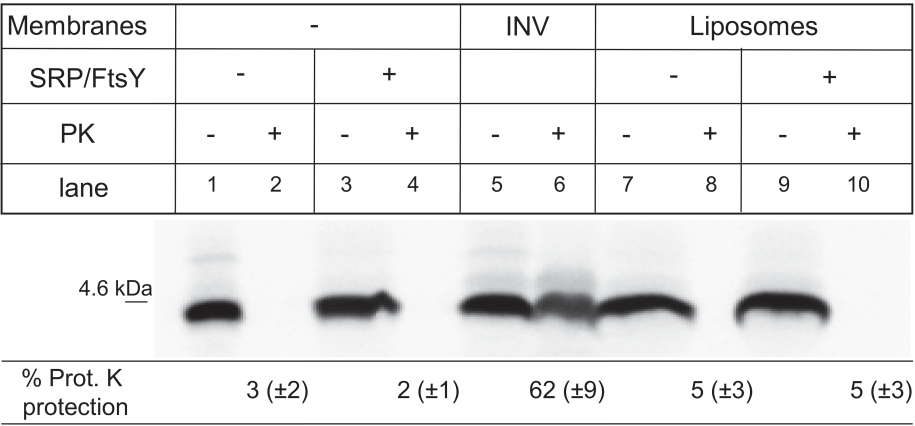

**B**

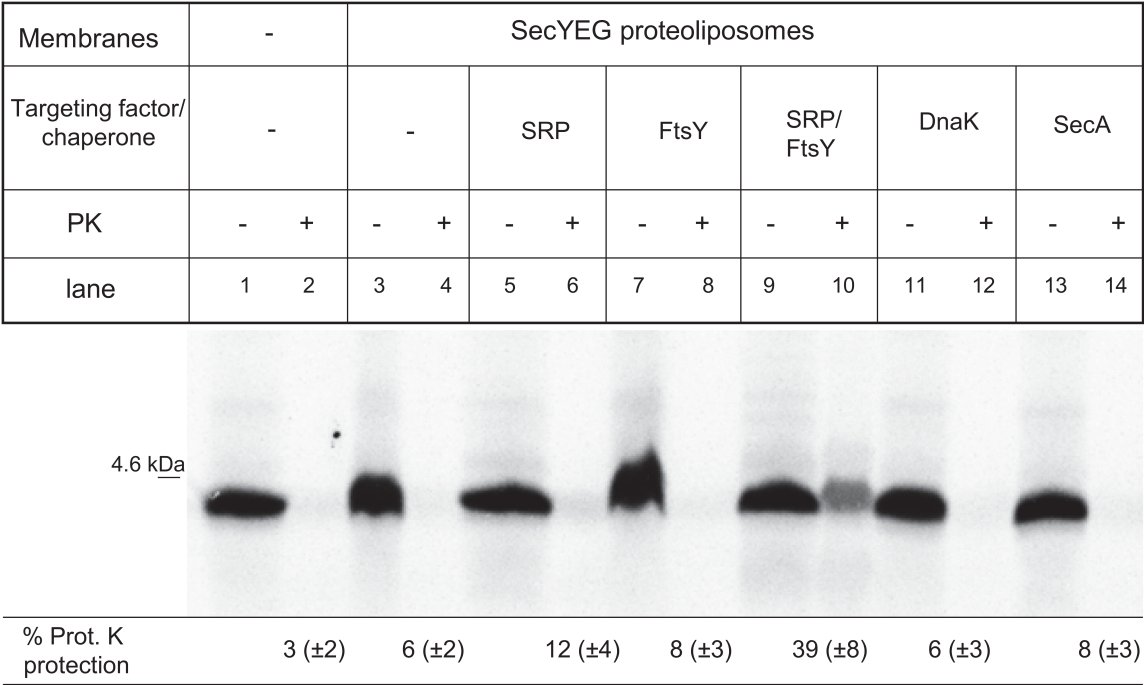

Supplement: S8 Fig — (A) YohP was in vitro synthesized in the absence of membranes, translation was stopped by the addition of chloramphenicol, and the sample was centrifuged for removing aggregates. The supernatant was subsequently incubated with INV or liposomes in the presence or absence of purified SRP/FtsY (20 ng/μl, each). Samples were then subjected to proteinase K treatment as before. Quantification was performed on three independent experiments, and the SEM is shown. (B) As in (A), but in vitro–synthesized YohP was incubated with SecYEG proteoliposomes in the presence of the indicated targeting factors/chaperones, which were present at a final concentration of 20 ng/μl together with a nucleotide mix (50 μM of each ATP and GTP in INV buffer). Quantification was performed on at least three independent experiments, and the SEM is shown. Underlying data for this figure can be found in S1 Data. INV, inner vesicle membrane; SEM, standard error of the mean; SRP, signal recognition particle. (PDF) [file pbio.3000874.s008.pdf]
