# Supplementary material for: Posttranslational insertion of small membrane proteins by the bacterial signal recognition particle
Source: PLoS Biol. 2020 Sep 30;18(9):e3000874. doi: 10.1371/journal.pbio.3000874 (PMC7549839; doi:10.1371/journal.pbio.3000874)
Supplement: Attachment — Submitted filename: Response to reviewers.pdf [file pbio.3000874.s011.pdf]

Dear Dr. Richardson,

We would like to thank you and the reviewers for the insightful and thorough evaluation of our manuscript. The reviewers identified some crucial limitations of our study, which we addressed in the revised manuscript. We have included many additional controls and additional in vivo data, which we think provide further evidence for an SRP-dependent post-translational insertion of small membrane proteins.

In respect to the *in vivo* data, we would like to mention that the original manuscript contained already proof for an *in vivo* interaction of YohP with SRP (now **new Fig. 5B**). We furthermore also included *in vivo* data on the localization of the *yohP* mRNA (**new Fig, 7**).

As to the use of conditional depletion strains *in vivo*, we would like to emphasize that the effect of *in vivo* depletion of SecYEG or YidC on the insertion of small membrane proteins was already determined by Fontaine et al., (2011). However, as we discussed in detail in our manuscript, those depletion experiments can be misleading and this is what we actually show in our revised manuscript **(new Fig. 6**).
For the same reason, *in vivo* SRP-depletion experiments can be misleading, because SRP-depletion also reduces the cellular levels of SecYEG and YidC, while proteases and chaperones are induced. This makes it difficult to assign any effect of SRP-depletion on small membrane protein insertion simply to a reduction of the essential SRP. Nevertheless, we have included a representative data set in our response to reviewer 3 and also discussed this issue further in our response.

The following new data have been included in the revised manuscript:

1. An electron microscopy evaluation of cells expressing the small membrane protein YohP, showing the lack of inclusion bodies (**new Fig. 1C**).

2. A cell fractionation study, showing that YohP is an integral membrane protein (**new Fig 1D**).

3. A biochemical analysis of the YohP topology, showing an N_{out}-C_{in} topology and further validating that YohP is membrane-integral and not just membrane-associated (**new Fig. 2C**).

4. In vitro transport assays, confirming the N_{out}-C_{in} topology and revealing the identity of the proteinase K protected fragments (**new Fig. 3C**).

5. Additional cross-linking experiments, which further demonstrate that SRP contacts small membrane proteins post-translationally, while SRP binds to longer membrane proteins co-translationally (**new Figs. 5 & S6**).

6. Proteoliposome studies, demonstrating that YohP can be inserted by both the SecYEG translocon and the YidC insertase (**new Fig. 6**) and that this is only achieved by the combined activity of SRP and its receptor FtsY. Other targeting factors or chaperones cannot support YohP insertion (**new Fig. S8**).

7. In vivo data showing that insertion of YohP into the bacterial membrane is initiated by an mRNA targeting step (**new Fig. 7**).

A point-to-point response to the reviewers' concerns is included below.

Thank you very much for your support and we apologize for our lengthy point-to-point response, but we wanted to clarify some issues that obviously were not sufficiently explained in our initial submission.

Sincerely yours,

Hans-Georg Koch

Reviews

Reviewer #1:

In this manuscript, Steinberg et al investigate the targeting and insertion mechanism for a class of small membrane proteins. The authors presented intriguing data to show that a 27 amino acid SMP, YohP, can physically associate with SRP, and the SRP pathway potentially participate in the targeting of YohP in a post-translational mechanism. These findings are interesting, as they raise the new possibility of a post-translational function of the SRP pathway. Nevertheless, the data presented do not yet rise to the standard of proof to make this rather extraordinary claim. A number of corroborating experiments and controls are needed, in addition to a significant tone-down of the conclusions and clarification of the interpretation.

Response:

- *We thank the reviewer for the support and suggestions.*

Figures 1 and 2 mostly aim to establish the in vivo membrane localization of YohP using fluorescence microscopy and fractionation experiments. However, neither experiments yielded convincing data. The localization of YohP-GFP is unusual and punctate; while there may be interesting explanations for this observation, these data do not show that YohP-GFP is membrane-localized. Rather, it seems to be in some type of inclusions, raising questions as to whether the microscopy data are are artifact of YohP overexpression, fusion to GFP, or both. The cellular fractionation experiments could not exclude the possibility that YohP is in the 'pellet' fraction because it is in the inclusion body, or aggregated (both are pretty common upon membrane protein expression). These experiments will need to be redone with more sophisticated fractionation protocols that separate the inclusion body, inner membrane and periplasm, and should include the appropriate control proteins with known cellular localizations.

Response:

- *We agree with the reviewer that the initial data set did not proof without any doubt that YohP is membrane localized.*
- *We have now analyzed YohP localization by electron microscopy (**new Fig. 1C**), which did not show any indication for the formation of inclusion bodies.*
- *We have furthermore performed thorough cell fractionation experiments, which validated the membrane localization of YohP and further demonstrated that YohP does not significantly accumulate in inclusion bodies/aggregates (**new Fig. 1D**).*
- *In the original Fig. 1D (now **new Fig 2B**) we better described the nature of the material that is displayed in this figure. Our original description obviously left the impression that we loaded simply a supernatant and pellet after cell breakage. However, we first generated a S30 cell soluble cell extract (hence, possible inclusion bodies were removed), which was then further separated into the cytosolic fraction and the membrane fraction. This is now better described in the revised manuscript.*

The AMS labeling experiments are much more specific and may provide stronger evidence for the membrane insertion of YohP. However, since this is a previously uncharacterized method, these experiments need to be supported by control data to show that AMS labeling worked as the author suggested, i.e., it is accessible to the bacterial outer membrane and periplasm, but not the inner membrane or cytoplasm.

Response:

- *Although AMS labeling has been frequently used for determining the orientation of membrane proteins in the bacterial membrane (e.g. Fujihira et al., 2002; Kimura et al., 1997; Neugebauer et al., 2012), we decided to remove these data from the manuscript.*
- *Instead we used another assay for validating the topology of YohP both in vivo (**new Fig. 2C**) and in vitro (**new Fig. 3A**). In contrast to our initial suggestion, these new data demonstrate a N_{out}-C_{in} topology. We currently don't know why the AMS labeling suggested another topology,*

but this will be followed up in future experiments. It is likely that inserting the cysteine residue required for AMS-labeling changes the topology of YohP.

The in vitro experiments to reconstitute YohP insertion into inverted membrane vesicles are significantly stronger. Additional experiments and clarifications are needed in this component. (i) The authors should show that YohP was inserted in the correct topology in the reconstituted insertion assay, either by repeating the AMS labeling experiment in the in vitro setting or use another method.

Response:

- *The topology of the in vitro synthesized and integrated YohP was now confirmed and shows the same topology as in vivo (**new Fig. 2C and 3A).***

(ii) The involvement of SecYEG in YohP insertion based on the putative inhibitor IpomF is interesting but inconclusive. The translocation of OmpA is dependent on both SecYEG and SecA; therefore, the inhibition of OmpA translocation could arise from IpomF inhibition of SecYEG, SecA, or another component in the assay. Either this inhibitor needs to be characterized with additional SecYEG dependent, SecA independent substrates, or an independent mechanism to inhibit SecYEG need to be used to establish the involvement of SecYEG in YohP insertion.

Response:

- *We agree with the reviewer that we cannot entirely exclude that IpomF also inhibits SecA or any other component of the bacterial protein transport machinery. However, our data show that SecA is not involved in YohP insertion **(Fig. 3B and new Fig. S8**), therefore a possible inhibition of SecA by IpomF would not explain the insertion defect for YohP that we observed in the presence of IpomF.*
- *The involvement of SecYEG in YohP insertion was further established by using reconstituted proteoliposomes **(New Fig. 6**), which demonstrate that YohP can be inserted SRP/FtsY-dependently via SecYEG or YidC.*

(iii) The role of supplemented SRP/FtsY in stimulating YohP insertion is most interesting. I am a little confused about this experiment, though, as the S135 extract the authors used for in vitro translation of YohP should contain all the targeting factors including SRP/FtsY. If the authors switched to a PURE in vitro translation system here, it was not described. If the authors were using the S135 extract, then this question needs to be resolved with experimental evidence. In addition, the authors need to perform PK digestion under +SRP/FtsY, -INV conditions.

Response:

- *We apologize to the reviewer that we did not make it clear enough that these experiments were performed with the purified in vitro translations system that is devoid of SRP/FtsY. We also included a control showing that the addition of SRP/FtsY in the absence of INV does not result in proteinase K protection of YohP **(new Fig. S8)**.*

The crosslink between YohP and Ffh is another piece of interesting data and provides evidence for a post-translational mechanism of SRP interaction with a membrane protein. I suggest a few more experiments to strengthen this component. (i) additional Bpa incorporation sites on YohP should be tested for crosslinking to Ffh. The current Bpa site (aa4) is near the N-terminus of YohP. With the additional His6 tag, the size of YohP is ~33 residues, placing the N-terminus at the vestibule of the exit tunnel; thus the current data could not exclude a cotranslational contact with SRP. There have recently been a number of reports showing that protein biogenesis factors including SRP could partially insert into the exit tunnel to enable potential contacts with the nascent chain. (ii) the puromycin release experiment in Figure 5 should include a control with a strictly cotranslational SRP substrate, and demonstrate that nascent chain is successfully released by puromycin treatment.

Response:

- *We agree with the reviewer that recent data have shown that SRP and SecA can partially insert into the ribosomal tunnel. Therefore, we cannot entirely exclude that the current pBpa site in YohP (amino acid 4) allows for a co-translational interaction with SRP. Still, the available data demonstrate that a stable co-translational contact between SRP and a nascent membrane requires at least 40 amino acids (Houben et al., 2005); 42 amino acids (Denks et al., 2017, Schibich et al., 2016) or 50 amino acids (Mercier et al., 2017). No SRP contacts to nascent substrates of less than 40 amino acids have been observed so far and no contact between SRP and membrane proteins of less than 50 amino acids were observed in ribosome profiling studies (Schibich et al., 2016).*
- *For providing a more definite proof that SRP recognizes its substrate post-translational, we have repeated the cross-linking experiment with a YohP variant that carried pBpa at position 27 (**new Fig. 5A**). We also included a control showing that the addition of puromycin blocks protein synthesis completely (**new Fig. 5A**). We furthermore included a control showing that the cross-link between SRP and ribosome-associated nascent chains of the classical co-translational SRP substrate MtlA was not observed in the presence of puromycin (**new Fig. S6**).*
- *Finally, it is important to emphasize that the data shown in new **Fig. 4A** clearly demonstrate that SRP acts at a post-translational step of YohP insertion.*

Finally, although the manuscript provides very interesting in vitro data to show that the SRP pathway could post-translationally contact and assist in the targeting and insertion of YohP, the specificity of the role of SRP (i.e., can any cellular chaperone do the same) and the in vivo relevance of SRP in YohP insertion have not been established. These components will be necessary to establish the new pathway that the author claims.

Response:

- *Demonstrating the specificity of the role of SRP in YohP insertion is a crucial point. In the original manuscript we had already shown that neither SRP nor FtsY alone can support YohP insertion, but that both are required for efficient insertion. We furthermore demonstrated that SecA does not support insertion, which we validated also in the new proteoliposome approach (Fig. 3 and **new Fig. S8**). We also tested purified DnaK in this assay and did not see any YohP insertion, further excluding the possibility that any chaperone can support YohP insertion (**new Fig. S8)**.*
- *By performing the cross-linking in vivo, we had already shown that the interaction between YohP and SRP is not an artefact, but also observed in living E. coli cells. We have now extended this further by also testing for a possible contact to the chaperone Trigger factor, but were unable to detect any contact (**new Fig. 5**), further validating that the in vivo contact between YohP and SRP is specific. We also confirmed SRP cross-links to YkgR, another small membrane protein (**new Fig. S6**).*
- *As to the in vivo relevance: see our comment and data set in response to the concern of reviewer 3 (page 9).*

Additional minor points:

The manuscript needs additional proofreading. For example, the end of line 1015 states, "The values are means of xx number of experiments". For another example, Figure 2B had only 8 lanes in the gel image but 9 labeled lanes. The labels and gel lanes are out of alignment, too.

Response:

- *We apologize for these mistakes and have corrected them.*

2. It is unclear why YohP is not completely digested in the absence of INV in Figure 3B, while it was in Figure 3A. In addition, the changes in the intensity of the membrane protected band in +INV reactions in the presence of IpomF (Figure 3B) correlate with the intensity of this full-length, undigested YohP band, raising concerns about whether the effects are mostly from variations in loading and/or proK digestion efficiency rather than ImpF inhibition of insertion.

Response:

- *Incomplete digestion is unfortunately frequently observed with YohP and other small membrane proteins and probably reflects the formation of proteinase K resistant aggregates. This problem was solved when in vitro synthesized YohP was centrifuged before the addition of INV, liposomes or proteoliposomes (**new Figs. 4, 6 & S8**).*

3. Related to point (2), most of the gels in the manuscript lack loading controls, making quantification (such as Figure 3C) problematic.

Response:

- *The reviewer obviously was under the impression that the data shown in the original Fig. 3 **(now new Fig. 6**) represent western blots and therefore asks for loading controls. However, these data show the insertion of in vitro synthesized and radioactively labelled material and therefore the quantification is reliable and does not require a loading control. This is now more clearly described this in the revised manuscript. We have also included more quantifications of the insertion of vitro synthesized YohP.*

Reviewer #2: Gisela Storz, signed review

In this study, Steinberg et al. examine the mechanism by which the 27 amino acid YohP protein is inserted into the membrane. Using GFP-, His- and AMS-tagged derivatives in vivo and translocation assays in which they block or deplete the activities of the signal recognition particle (SRP), SecYEG and associated proteins in vitro, the authors conclude that YohP is translocated posttranslationally by SRP together with FtsY and SecYEG but independent of SecA.

The study is clearly written and the in vitro assays, particularly the approach of using Ipomoeassin F, are clever. However, I have the following comments:

Response:

- *We thank the reviewer for the encouraging comments and suggestions.*

1. Several statements are too general and need to be modified to better reflect the results:
--Page 2, Lines 29-30: "our data reveal that SMPs engage a unique post-transcriptional SRP-dependent targeting pathway…". The authors have only carried out experiments for YohP.

Response:

- *We agree with the reviewer and have toned down our statements. We also included YkgR as another small membrane protein in the cross-linking approach and also found that it post-translationally interacts with SRP (**new Fig. S6**)*

--Throughout: Given that the authors do not have an in vivo assay for YohP function, they do not know whether even a small tag (AMS or His) might affect its function (as has been found for other small membrane proteins (as in PMID 28512220).

Response:

- *The reviewer is correct in stating that we do not have an in vivo assay for YohP function (which currently is the case for most small membrane proteins). Therefore, we cannot entirely exclude that the addition of the His-tag affects YohP function. However, we would like to emphasize that we used different experimental strategies: immune detection of YohP-His, in vivo radioactive labelling of YohP and YohP-His and in vitro radioactive labeling of YohP and*

*YohP-His. In these assays we did not observe any indication that the His-tag influences insertion and/or topology (**new Figs. 1, 2, 3, 4, 5 & 6**)*
- The AMS data were removed from the manuscript.

--Page 11, Line 222 (and elsewhere): "YohP insertion is, like OmpA translocation, dependent on the SecYEG translocon". This has only been shown in vitro for this study.

Response:

- *Again we agree with the reviewer. In the revised manuscript, we have included additional data on the SecYEG- and YidC-dependency of YohP insertion **(new Fig. 6)**.*

2. Other experimental suggestions:
--Page 10, line 187: "…presumably lacking a few N-terminal amino acids". This should be determined since it has implications for comments about YohP orientation in the membrane.

Response:

- *We have now compared the in vitro insertion of His-tagged and non-His-tagged YohP and our data demonstrate that the size shift is due to cleavage of the His-tag (**new Fig. 3A**).*

--Throughout: Since some of the effects are small, it would be useful to have some sense of the reproducibility of the results. For example in Figure 2B, why are the levels of YohP2 lower at 0.5 mM IpoMF than for the 0 and 1.0 mM treatments.

Response:

- *We have included for all data sets a reproducibility statement and have indicated how often experiments were reproduced. For the quantification of YohP insertion we did not include the dimeric YohP band because its appearance in the in vitro assays did not always correlate with the amount of the monomeric YohP band. The reason for this is currently unclear and we have mentioned this in the revised manuscript.*

3. Other editorial comments:
--Throughout: Since the small protein field is full of confusing nomenclature, I suggest eliminating the use of SMP.

Response:

- *We have eliminated the term SMP and replaced it with the term small membrane proteins*

--Page 10, line 188-189 (and elsewhere): "We also tested YohP insertion into liposomes composed of E. coli phospholipids." The authors should be very clear about what components are in each of these assays.

Response:

- *In the revised manuscript, the composition of the assays systems is more clearly described both in the Material and Methods section and in the text.*

Reviewer #3:

Steinberg et al, investigated possible routes for targeting and insertion of small membrane proteins. During translation, these proteins are buried inside the ribosomal polypeptide exit channel, and leave

the ribosome only at the end of their translation. This might prevent co-translational interaction with chaperones and enzymes that usually associate with exposed nascent chains, including the SRP.

However, this concern is not fully justified, because there have been indications that the SecA and SRP can interact with such ribosomes before the exit of nascent chains (Bornemann et al, 2008; Chartron et al, 2016; Mercier et al, 2017; Denks et al, 2017; Knüpffer et al, 2019). As proposed, such interactions allow SRP to scan ribosomes very early for potential substrates, even before the hydrophobic polypeptide is fully exposed from the ribosome.

Response:

- *We completely agree with the reviewer, also because two of the studies the reviewer mentioned are actually from our group (Denks et al 2017; Knüpffer et al., 2019). Still, it is important to differentiate between contacts of SRP to a translating ribosome and contacts of SRP to the nascent polypeptide. While SRP can contact the ribosome before the polypeptide is fully exposed, stable contacts to the polypeptide are only observed when the chain reaches a length of more than 40 amino acids (Denks et al., 2017; Mercier et al., 2017; Houben et al, 2003; Schibich et al, 2016). This is also validated through in vivo experiments (Noriega et al., 2014). In the revised manuscript we have more clearly described this.*
- *However, to exclude the possibility that SRP makes stable contact to YohP within the ribosomal tunnel and to validate our results that the YohP-SRP cross-link is also observed after dissociation of the ribosome by puromycin (**new Fig. 5)**, we included additional experiments: (1) We included a control SRP substrate (mannitol permease) and demonstrated that SRP contacts to nascent chains of mannitol permease were in contrast to YohP puromycin sensitive (**new Fig. S6**). In addition, we incorporated the cross-linker at the C-terminus of YohP and still observed cross-links to SRP. Finally, we also tested another small membrane protein, YkgR, and also found puromycin-insensitive cross-links to SRP (**new Fig. S6**). In our view these new data clearly demonstrate a post-translational interaction of YohP with SRP.*

Previous studies suggested that the biogenesis of one such small membrane protein in yeast requires translation-independent targeting of its encoding mRNA to the ER membrane. However, the question whether the SRP system is involved in the pathway has not been studied (Loya et al, 2008, RNA). Other studies have suggested that even in that case, the SRP is important (Chartron et al, 2016). Theoretically, nevertheless, even if mRNA targeting is translation-independent, it does not rule out a possible involvement of the SRP at a later stage of the biogenesis of this yeast small membrane protein, which may occur on the ER membrane.

The authors of the present work may decide to discuss the above raised issues, which seem very relevant, more thoroughly.

Response:

- *This is a very valid point that prompted us to analyze the localization of the YohP mRNA in vivo in E. coli cells. Our data show that the YohP mRNA preferentially localized to the membrane, while the mRNA of the cytosolic protein BglB was localized in the cytosol (**new Fig. 7)**. These in vivo data suggest a translation-independent targeting of the YohP mRNA that is followed by the post-translational and SRP-dependent insertion.*

Regardless of the above-mentioned issues, the authors investigated thoroughly, both in vivo and mainly in vitro, several aspects of the proposed post-translational targeting and insertion of a single small membrane protein, YohP, in E. coli. Based on the results, an SRP-dependent post-translational targeting pathway is proposed. Although the work began with four putative small membrane proteins (Fig. 1A), it was finally focused on a single protein YohP. The authors do not explain why another possible candidate, YkgR, has been omitted (In which case, Fig. 1A may be unnecessary). The authors propose that their work on a single protein, YohP, is representative: "our data reveal that SMPs engage a unique post-translational SRP-dependent targeting pathway."

Response:

- *We agree with the reviewer that our statement might be to general and we have toned down the statements. We have also included additional data on YkgR, which show that SRP also cross-links to YkgR **(new Fig. S6)**.*

When considering the authors' suggestion that YohP biogenesis occurs post-translationally and is mediated by the SRP system, several questions arise: Where does this happen and what is the mechanism? These issues were investigated by in vitro experiments, which show relatively convincingly that the PK cleavage of YohP in the presence of inverted vesicles can be prevented by the SRP system (suggesting membrane integration). This was reproduced by using separate preparations of in vitro synthesized YohP and SRP components. Whether such a pathway operates in vivo remains to be investigated, possibly by utilizing depletion strains. Another mechanistic aspect of the proposed pathway is whether YohP interacts with the SRP. This has been shown convincingly both in vitro and in vivo. It would be interesting to see whether the SRP is able to interact with unrelated hydrophobic polypeptides post-translationally, under the same conditions used in the present work (as "negative"controls).

Response:

- *Determining the SRP-dependency of YohP insertion in vivo is a critical issue and we demonstrate that YohP is cross-linked to SRP in vivo* **(new Fig. 5)**.
- *The request for a negative control for cross-linking is valid. However, there could be multiple reasons for not observing a cross-link and therefore we rather executed additional controls: (1) We performed the cross-linking experiment with another small membrane protein, YkgR, and observed also an SRP-YkgR cross-link (**new Fig. S6**). (2) We repeated the in vivo cross-link and tested for contacts between YohP and the ribosome-bound chaperone trigger factor; here we were unable to detect any specific cross-link **(new Fig. 5**). (3) Finally, we analyzed the SRP cross-link to the classical SRP substrate mannitol permease and demonstrated that this contact is strictly co-translational, in contrast to the SRP-YohP cross-link (**new Fig. S6**).*
- *We also determined the YohP insertion in vivo by using a conditional Ffh-depletion strain. Upon Ffh depletion we observed reduced in vivo synthesis of YohP. Furthermore, most of the in vivo synthesized YohP was present in the P30 fraction after cell breakage (**see figure below**), which indicates the formation of inclusion bodies/aggregates. These data support our conclusion that SRP is involved in YohP insertion. However, because SRP-depletion causes many secondary effects, it is impossible to determine whether the observed effects are just caused by SRP depletion or (additionally) by secondary effects, like reduced SecYEG/YidC-content, increased proteolysis, increased chaperone production or reduced translation. All these effects have been observed upon SRP depletion. Therefore, we prefer to not show these data, because we cannot reliably assign the effects to simply Ffh depletion.*

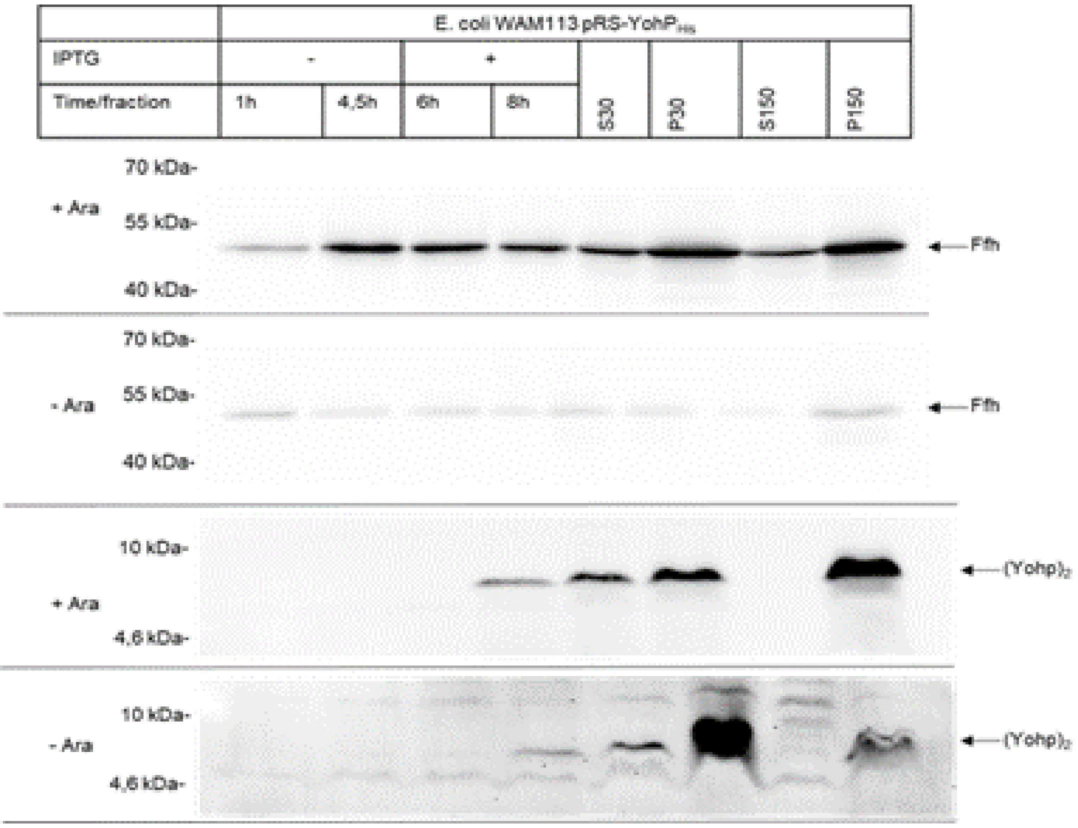

Figure for review: *The conditional Ffh (SRP)-depletion strain Wam113 containing the YohP expression plasmid pRS-YohP$_{His}$, was grown in the presence (+ Ffh) or absence of arabinose (-Ffh). IPTG for induction of YohP was added after 6 hours of growth and cells were fractionated after 2 additional hours of growth. Indicated is the soluble cell extract after cell breakage and centrifugation (S30) and the corresponding pellet fraction (P30), containing unbroken cells, large aggregates and inclusion bodies. The S30 extract was then further separated by centrifugation in the cytosolic fraction (S150) and the crude membrane fraction (P150). Fractions were decorated with α-Ffh antibodies (upper two panels) or α-His antibodies (lower two panels). Please note that the lowest panel, i.e. expression of YohP upon Ffh-depletion required significantly longer exposure for detection, indicating a much lower expression or faster degradation of YohP upon SRP-depletion.*

Comments

1. It is shown convincingly that YohP appears in a non-soluble material. The authors conclude that it is in the membrane fraction (likely correct, but does not exclude some aggregation). Maybe using flotation would confirm that it is in the membrane.

Response:

- *We have used more thorough cell fractionation methods for validating the membrane localization of YohP (**new Fig. 1 & 2**), which clearly show that YohP is localized in the inner bacterial membrane in a carbonate-resistant state.*

2. The authors suggest that some of the YohP protein is degraded during fractionation. This is not a major issue. If it was, the authors could easily test it by various incubation times after cell disruption.

3. **Response:**

• *We agree with the reviewer and have toned down our statement.*

4. Although the authors dedicated part of their work to the question how YohP dimerizes, it is not directly related to the main research goals. Maybe the authors would agree to skip this part because of the following: The results do not really prove dimerization, because they may reflect SDS-PAGE artifact, which happens frequently with hydrophobic membrane proteins. One cannot exclude that the mutations may have prevented the artifact. If the authors want to detect true dimerization, they may consider using use native gels, co-precipitation with different tags, etc.

Response:

- *We agree with the reviewer that the dimer issue is less important for the main conclusion of the manuscript. However, as we frequently see the dimeric band in our gels, we think we cannot completely ignore it and we therefore kept the data but moved them into the supplement (**new Fig. S4)**.*

5. In contrast to the dimerization issue, testing the orientation (and actually the full membrane crossing) of YohP in the membrane is important. The results show convincingly that the C-terminus of YohP is externally exposed (Fig. S3, the word "membranes" should be replaced by the word "pellets").

Response:

- *The AMS-labeling data and Fig. S3 were removed from the manuscript, because the new data indicated that the majority of YohP is oriented with a C_{in}-topology both in vivo and in vitro. The reason why the AMS labeling suggested an inverse topology is currently unknown, but will be followed up in future experiments.*
- *We have verified that YohP is an integral membrane protein by showing its enrichment in the inner membrane vesicles (**new Fig. 1D**) and is carbonate-resistance (**new Fig. 2C**).*

5. The authors tested whether the eukaryotic Sec61[illegible] inhibitor IpomF also inhibits E. coli SecYEG. The results suggest at least partial inhibition, although the mechanism is not known. Notably, the amount of applied IpomF was substantially higher than that used in the eukaryotic system. The results with OmpA signal sequence suggested to the authors that IpomF does not prevent initial association with the SecYEG, thus leaving the question why the inhibitor affected YohP insertion unanswered. The authors prefer IpomF, over depletion studies (SecE-depletion) because of possible indirect pleotropic effects. This may be partially true in some cases, though numerous aspects in cell biology have been revealed by depletion experiments. In any case, the use of inhibitors in combination with genetic depletion may be a good choice, especially when the inhibitor does not seem to perform as would have been expected (concentration wise). The authors' conclusion from these studies is interesting and intriguing in light of previous suggestions that YidC is important for biogenesis of several small integral membrane proteins. Previous work on YohP (Fontain et al, 2011) suggested promiscuous requirements for several small membrane proteins, mainly based on depletion studies.

Response:

- Again, we agree with the reviewer that our initial data with IpomF did not exclude that YohP can also be inserted by YidC. Fontaine et al., (2011) actually concluded based on results using conditional SecE or YidC depletion strains that YohP is inserted independently of the SecYEG translocon or YidC. However, as we have stated in our manuscript the interpretation of these results is difficult because some membrane proteins can be inserted by either SecYEG or YidC. We therefore used reconstituted proteoliposomes for demonstrating that indeed YohP can be inserted by either SecYEG or YidC (**new Fig. 6**). This is in agreement with the in vivo results from Fontaine et al., although the interpretation is different.

6. Row 256-258, "YohP was synthesized in vitro in the absence of membranes and translation was then stopped by the addition of chloramphenicol, followed by centrifugation to remove ribosomes and any potential protein aggregates." Here the authors suggest that the very hydrophobic YohP protein remains soluble! This is rather spectacular in itself: Hydrophobicity plot

Response:

- *This is a valid point and we would like to emphasize that by performing this experiment we indeed remove a substantial amount of YohP, which is likely aggregated in the absence of membranes. Nevertheless, the supernatant still contains enough YohP to perform the subsequent experiments. This is an advantage of the in vitro system, because the sensitivity of detection method (radioactivity) is so high. We would also like to mention that although the purified in vitro system is free of SRP/FtsY, it still contains chaperones, which could keep YohP in solution. We have specified this in the revised manuscript.*
- *A hydrophobicity plot is shown below, but we don't think it is necessary to include this in the manuscript.*

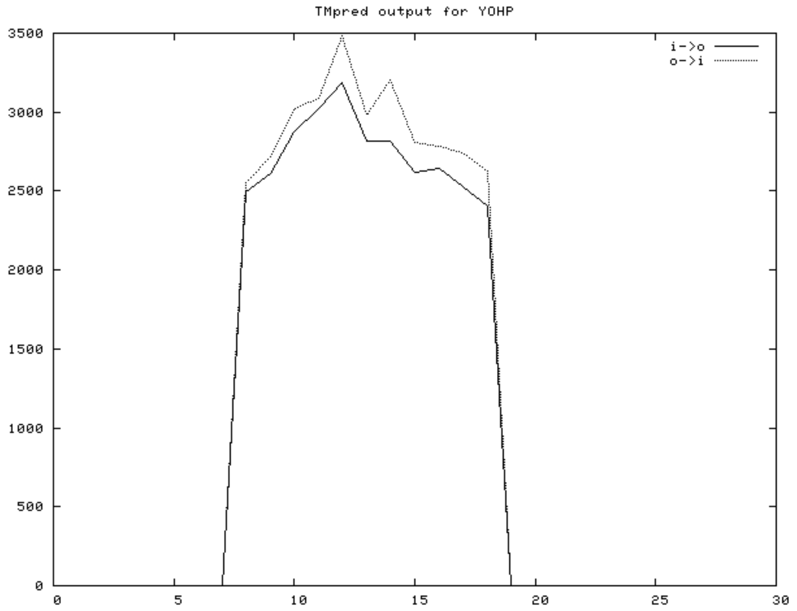

Minor comments:

1. What is the difference between purified in vitro system and the indicated S-135?

   Response:

   - *The difference between the two in vitro systems is now more clearly described. The purified system lacks targeting factors like SecA and SRP and is generated by sucrose density centrifugation of a soluble cell extract with subsequent fractionation. Fractions were then tested for in vitro translation activity and the absence of SRP, SecA and FtsY. The purified cell extract was used for all in vitro experiments, with the exception of the cross-linking experiments, because in these experiments we did not want to bias the results by removing any targeting factors or chaperones.*

2. Row 214, "either" can be deleted.

   Response:

   This was deleted.

3. Introduction- make clear that SRP=Ffh+4.5S RNA

Response:

This was included

4. Row 281, "were" purified (instead of "was").

Response:

This was corrected

5. Figures

Fig 2B, the legends do not correspond to the lanes (alignment is needed).
Fig 4A, should have shown SecYEG overexpression.
Fig 4C, how many repeats (xx)?
Fig 5A: the IP does not show controls (eg -Ab).

Response:

- *This has been corrected or additional information has been added. We did not include a control for the IP, because the in vivo crosslinking clearly demonstrates that the cross-link observed reflects a SRP-YohP cross-link.*
- *The SecYEG expression levels of the INV used in these experiments have been demonstrated (Petriman et al., Scientific reports, 2018)*
- 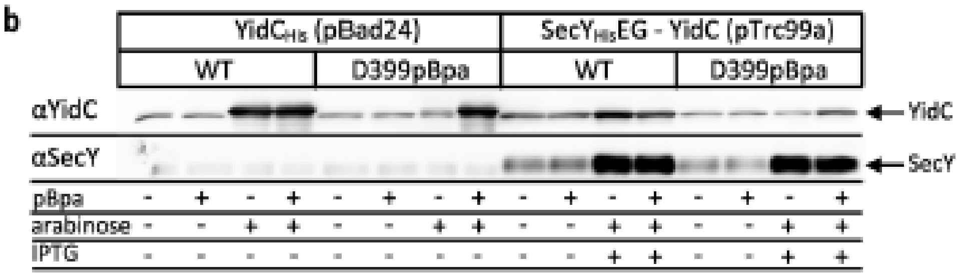

  C-terminus of YidC, which have not been crystallized so far. (**b**) The co-expression system shows balanced expression of YidC and SecYEG as revealed by western blotting. 1 × 10^8 *E. coli* BL21 cells expressing *yidC* under the arabinose promoter from plasmid pBad24 or co-expressing P_{lac}*secYEG-yidC* from plasmid pTrc99a were TCA precipitated, separated by SDS-PAGE and after western transfer decorated with the indicated antibodies. WT refers to wild type YidC and D399pBpa to a YidC variant with pBpa inserted at position 399, when pBpa was added to the growth medium. (**c**) *In vivo* photo-cross-linking performed with BL21 *E coli* cells

5. The term SMP (for small membrane proteins) is the same as that coined for Synaptotagmin-like mitochondrial-lipid-binding (SMP). This proposed SMP term for small membrane proteins has not been used by the researchers who identified many of them.

Response:

- *We replaced the term SMP with small membrane protein(s).*

7. western should be Western.

8. Row 104, maybe membrane protein (instead of proteins).

Response:

- *Both errors have been corrected*

9. Row 258, centrifugation, what are the conditions (g, time, temp). This is not always clear from the methods section.

Response:

- *This has been added in the revised manuscript.*

Reviewer #4:

Steinberg et al present an important novel observation about the pos-translational role of SRP in interacting and mediating membrane-targeting of small membrane proteins.

Response:

- *We thank the reviewer for the support and suggestions.*

Despite the importance of the study I have detailed below several suggestions for consideration in a revised version. These changes are important to solidify the observations and for clarity for the reader. The way the experiments are shown now I find that in some cases they do not strongly justify the statements that the authors want to make. Briefly:
a. I find that the MS suffers from not systematically quantifying the reactions that are being analyzed and hence these lead to largely qualitative statements, making it difficult to safely compare across conditions and draw firm conclusions about the intensity of the events.

Response:

- *Wherever possible, we have included quantifications*

b. There are multiple controls missing in various experiments including definitively shown the role of SecY or absence of contribution of YidC, defining a simple probing of whether SRP is only mediating targeting or is involved in a downstream reaction etc.

Response:

- *We have included several in vitro and in vivo experiments for addressing these questions as detailed below.*

c. The presentation and manuscript organization would benefit from some trimming-rearrangements that I have detailed below (e.g. discussion could be trimmed, Fig. 1A contains unnecessary molecules etc), the authors should direct the reader more precisely to the data etc.

Response:

- *The manuscript has been reorganized for focusing more on the central data.*

d. They developed a powerful in vitro system that they are underusing.

Response:

- *In the revised version, we added several additional in vitro experiments, in particular we have used proteoliposomes for validating that YohP can be inserted by either SecYEG or YidC (**new Fig.6**). We furthermore demonstrate that the combined action of SRP/FtsY is required for YohP insertion and that neither SecA nor DnaK are able to support YohP insertion **(new Figs. 3, 6 & S8)***

Specific points

1. These small proteins are referred to as SMPs. Is it clear that they fully integrate in the membrane? It would be a simple experiment in Fig 1A and D to treat the membrane

extracts with a chaotrope or high ionic strength and clarify this.

Response:

- *We have included addition experiments that show that YohP is highly enriched in the inner bacterial membrane and carbonate resistant (**new Fig. 1D & 2C**).*

2. If the authors are not interested in AzuC (and it does not have a predicted TM) and YshB is not synthesized in vivo and they focus on YohP, perhaps Fig. 1 should be in the supplement?

Response:

- *We have included now also YkgR in the cross-linking approach and show that it is also post-translationally recognized by SRP (**new Fig. S6)**.*

3. The YohP-GFP fusion topology studies seem to contain large "inclusion bodies" secluded to the poles by the chromosome and on top of these large bodies, some low intensity foci that may be peripheral. Have these experiments been done with lower induction of expression and at lower temperature such that if the large inclusion bodies result from over-expression they can be eliminated/reduced? Has the topology analysis been done using some objective numerical analysis of particle distribution in the cell? The authors correctly discuss in the next paragraph the possible interference coming from add on sequences particularly to such a small protein. How is it excluded here that the sub-cellular distribution seen is not affected by GFP?

Response:

- *We specified in the manuscript that we cannot make any conclusions about the subcellular distribution of YohP-GFP beyond the fact that it is membrane localized, because we don't know whether the GFP influences clustering in the membrane. This was also the reason to use tag-free approaches both in vivo and in vitro. We therefore feel that the reviewers concern was already addressed in the previous version.*
- *We agree with the reviewer that the punctate appearance of YohP in whole cells could indicate the formation of inclusion bodies. However, fluorescently labelled proteins trapped in inclusion bodies often lose fluorescence; this is e.g. seen in the YchF-GFP sample in Fig. 1, where dark spots appear at the cell poles. It is also important that YohP was expressed at levels that barely allowed the detection of YohP-GFP by immune-detection.*
- *We have included electron microscopy pictures of cells expressing YohP and we don't see the accumulation of inclusion bodies **(new Fig. 1C)***
- *We have also performed more thorough cell fractionation experiments, which further validated the membrane localization of YohP (**new Fig. 1D**).*

6. Ln99-100 and Fig. 1C. It is not clear to me if the dimer could be attributed to an oxidized disulfide from Cys2. Have the samples been extensively reduced prior to electrophoresis, e.g. 10mM DTT?

Response:

- *Wild type YohP does not contain a Cysteine at position 2, this was only engineered for the AMS labeling experiments. This is now clarified in the text and in Fig. 1. We apologize for the error in in the original Fig. 1A.*

7. Fig. 1D. The vector control here is not very helpful as it hardly synthesizes anything. I think a membrane unrelated protein should be included to show opposite fractionation to that of YohP. Also, have the signals been properly quantified?

8. **Response:**

- *In our view the vector control is important because it demonstrates that the addition of rifampicin indeed blocks almost completely E. coli RNA polymerase, while T7-polymerase dependent transcription is still working in the pulse-chase experiments. Nevertheless, we agree with the reviewer that more definite proof of membrane localization is needed and we provide this by cell fractionation studies **(new Figs. 1D and 2C**). In the revised manuscript, we also provide quantification.*

9. Ln128 …in line with the presence of a transmembrane domain… In my opinion this statement is not accurate based on the microscopy.

Response:

- *The cell fractionation studies and the in vitro membrane integration assays support in our view clearly the membrane localization of YohP (**new Figs. 1D, 2B, 2C, 6**).*

Figs. Plz indicate lanes in the text when referring the reader to figures.

Response:

- *This has been included for the more complex figures.*

6. Fig. 1D. Given that this product comes from a T7 promoter and such in vitro transcription/translation systems are readily available, have the authors tried to see if addition of membranes to an in vitro synthesized YohP would allow membrane association??

Response:

- *This is shown in **new Figs. 4 and 6**.*

9. Line 144. The statement about stability in the membrane cannot really be made if the issue of reduced synthesis is not excluded first.

Response:

- *As reviewer 3 pointed out, the issue of YohP dimerization is less relevant for the central conclusion of the manuscript and we therefore moved these data into the supplement.*

10. In many Figures, gels and panels are misaligned

Response:

- *We apologize for not properly aligning gels and panels and have corrected this in the revised manuscript.*

11. Lines 147 and 148. Why then is the gly-gly motif approach used? The two glycine mutations may represent what was historically done but I think it is now misleading. In contrast to the double mutant, one would conclude that affecting the mobility of G15 prevents dimerization without affecting stability, while, in contrast to what is stated by the authors, G21 clearly promotes stability of the dimer or promotes instability only of the monomer.

Response:

- *We are not entirely sure that we understand the reviewers comment correctly. The importance of glycine zipper motifs for transmembrane domain dimerization has been established in multiple studies. It has also been established that although GxxxG is statistically the most over-represented dimerization motif found in membrane proteins, similar motifs were also found to be*

over-represented in membrane proteins. This was the rationale for analyzing the contribution of both glycine residues to YohP dimerization.

- *Our data clearly show that replacing Gly15 prevented YohP dimerization, while replacing Gly21 still allowed YohP dimerization. Whether this is the result of increased stability of the monomer in the Gly15 mutant and increased stability of the dimer in the Gly21 mutant, as the reviewer suggests, was not further analyzed in our study and is in our view beyond the scope of our manuscript. The only conclusion we drew from our studies is that Gly15 is important for dimerization and we think that this conclusion is still valid. Nevertheless, the YohP dimer issue is not in the centre of our manuscript and we have moved these data into the supplement.*

12. Line 158. What is then shown in fig. 1A, right is a cys mutant YohP.

<u>Response:</u>

- *Indeed, Fig 1A showed the cysteine mutant of YohP; in the revised version we show the wild type sequence for preventing confusion.*

<u>Response:</u>

13. Fig. 2D. the C2 mutation appears to reduce the amount of monomer and makes the dimer disappear. I don't think that one can really talk about accessibility by AMS or not, particularly if the authors are correct in their suspicion that the dimeric form is stabilized in the membrane. Also, there is no control to indicate that the membrane is intact and that protein of very well known topology with a cytoplasmic or a periplasmic cysteine would be labelled as expected in this assay.

<u>Response:</u>

- *We agree with the reviewer that the expression level of the C2 mutant is low, which could influence the interpretation of the AMS data. We therefore have removed the AMS data from the manuscript and have included alternative methods to determine YohP topology in vivo and in vitro **(new Figs. 2 & 3**).*

14. Line 171…or that the dimer flips both Cys towards the cytoplasm and away from AMS.

<u>Response:</u>

- *See above, these data were removed*

15. Fig. 3A.
a. Going back to a previous question I asked. Is the protein made in vitro soluble?? Have the membranes been added after synthesis? Does interaction with the membranes display kinetics? Does it require temperature?

<u>Response:</u>

- *The protein made in vitro is partially soluble and the membranes have been added after synthesis; this was shown and described in the original manuscript (**now new Fig. 4A and Fig. 6**). The kinetics of time-dependent membrane insertion of YohP at 37°C was further analyzed and the quantification (n= 2) is shown below:*

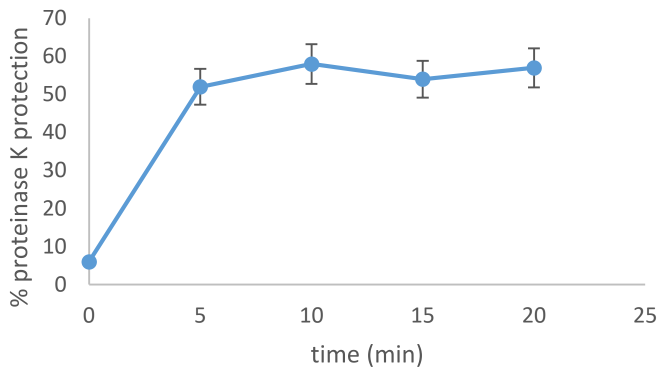

- *We also tested the temperature dependent insertion of YohP for 10 min and the quantification is shown below (n = 2):*

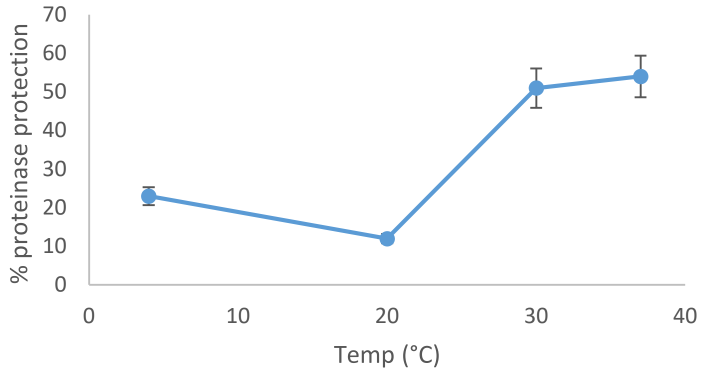

- *We opted to not include these data in the manuscript for the following reasons: (1) at low temperature proteinase K protected YohP aggregates are formed and therefore the quantification might be misleading. (2) YohP insertion into the membrane occurs very fast and even a 5 min incubation step with the membrane is sufficient for almost complete insertion. Shorter time periods are difficult to handle reproducibly in the in vitro translation/insertion system. (3) Kinetic analyses should be based on exact concentrations of the reaction partner. However, the in vitro synthesized material is impossible to quantify and is largely sub-stoichiometric to SecYEG/YidC and other components. We therefore don't feel that the incorporation of the data in the manuscript would adhere to basic concepts of protein kinetics, although the data indicate that YohP insertion is influenced by time and temperature in our in vitro system.*

b. Shouldn't proteinaseK degrade completely such a small protein cutting after hydrophobics? The explanations provided by the authors can be easily tested/excluded with a 400000g+ ultracentrifugation of their S135 after synthesis.

Response:

- *Protease resistant aggregates of small hydrophobic membrane proteins are expected and indeed when YohP was in vitro synthesized in the absence of membranes, we observed aggregates that could be pelleted by centrifugation (140.000 x g). The supernatant contained sufficient amounts of soluble YohP to monitor insertion after membranes were added. This is demonstrated in* ***new Figs 4, 6, & S8*** *in which aggregates were removed by centrifugation before membrane were added. After centrifugation, no proteinase K protected fragments were visible in the absence of membranes/liposomes.*

c. All experiments need to be quantified and normalized accordingly. For example, in this figure in lane 3 there are 2-3 times more starting material than in lane 1. If the PK-treated lane 2 was loaded 3 times more than the remaining monomer bands (the so called 3 PK-protected species) would be

very prominent and some remaining dimer might become visible. Before such a treatment, the conclusion in lines 184-186 is not really valid.

Response:

- *All membrane insertion assays have been quantified and the data are provided together with the number of technical/biological repeats.*

d. For the same reasons as in "c", the conclusions in lines 189-191 also do not seem valid, since lane 5 is also underloaded. Also, to demonstrate SecY or YidC dependence a more specific assay is needed. Either include SecY in the liposomes or block access to SecY in membranes? I think this is an important point to be made.

Response:

- *We have performed the requested quantifications and determined the proteinase K resistant amount of YohP under the different conditions. We also performed additional experiments for demonstrating the SecY-dependence of YohP-insertion (**new Figs. 6 & S8**).*

e. Have the Gly15 and 21 mutants been tested in this assay as they seem to predominantly favor one or the other state?

Response:

- *These mutants have not been tested in vitro, because all the available in vivo and in vitro data demonstrate that YohP is inserted into the membrane. As this reviewer and also reviewer 3 consider the data on YohP dimerization less relevant for the main conclusion, we opted against using this particular Glycine mutant in the in vitro assays.*

16. Fig. 3B and C.
a. Is IpomF an inhibitor of bacterial secretion in vivo? Is there more evidence here that what is observed with a molecule at 4mM (!!) results from specific inhibition of SecY? If this is not a very well characterized mechanism it is dangerous to use it as an internal control.
b. proOmpA gets processed and presumably membrane associates and becomes protected from proteinaseK. Is SecA and other such factors present in the S135 of this reaction?
c. As asked previously, is this apparent membrane integration time-dependent?

Response:

- *To our knowledge, IpomF has not been tested on bacteria in vivo. In vivo effects of these inhibitors are often not visible because these inhibitors are unable to cross the outer membrane. The specific inhibition of SecY is reasonable because the inhibitor binds to and inhibits also the eukaryotic homologue Sec61α. It is a general and so far unexplained phenomenon that inhibition of the bacterial SecYEG complex requires higher inhibitor concentrations than those required for the eukaryotic Sec61 complex (e.g. Junne et al., 2015).*
- *The SecY-dependency was further validated by additional proteoliposome experiments (**new Fig. 6 & S8**).*
- *SecA is added to the in vitro reaction by the INV. The in vitro synthesis was performed with the purified CTF system and this is now more clearly described in the manuscript. We cannot exclude that IpomF also inhibits SecA, but this would not explain the reduced YohP insertion in the presence of IpomF, because we clearly show that SecA is not required for YohP insertion **(new Figs. 3, 4, 6, & S8)**.*
- *Time-dependency of YohP insertion has been analyzed and it occurs very fast. Even after 5 min incubation with membranes, basically all YohP is inserted, **see above***

17. Lines 205-207. Do the authors know how uniform their IMVs are?

Response:

- *We don't completely understand what the reviewer is asking for. The INV isolation procedure follows an established protocol that we and others have used for many years. It is based on sucrose gradient centrifugation and therefore all INVs have roughly the same density. There might be some variation in size and there might also be a small population of right-side out vesicles. However, we don't think that this questions in any way our results and conclusions.*

18. Choice of MtlA complicates the analysis. Arent there any substrates that are striclly SecY- and strictly YidC-dependent to use?

Response:

- *OmpA is a strictly SecY-dependent substrate. MtlA was used because it can use either SecYEG or YidC for insertion and is also strictly SRP-dependent, but does not require SecA. Therefore, in terms of SRP-dependency and SecA-independency it behaves exactly like YohP. The newly established proteoliposome studies further establish the YohP insertion mode* ***(new Figs. 6 & S8)****.*

19. Fig. 4A. In the absence of quantification, the differences seen can be easily attributed to just loading differences (e.g. between lanes 3 and 4 and 5 and 6, since 5 has ~2x more material than lane 3) and not to SecY over-expression.

Response:

- *We have included quantification of all in vitro transport data.*

20. Fig. 4B and C. How much is added of each factor in molar terms? Have they been titrated? Is there a time and temperature dependence? Is GTP needed for the SRP to work? The number of repeast in the legend is mentioned as "xx".

Response:

- *The concentration of SRP (20ng/µl)/FtsY (20ng/µl)/SecA (80 ng/µl) that were added in these experiments was listed in the Material and Methods part and we have now included this information also in the legend to the Figures. We titrated different amounts of SRP/FtsY and SecA when optimizing these experiments and then used a concentration that was also used in previously published studies defining the SRP/FtsY- or SecA dependency of substrates (e.g. Braig et al., 2009; Welte et al., 2009; Koch et al., 1999, Denks et al., 2017)*
- *As to the GTP requirement: SRP and FtsY do not require GTP to interact with their substrates and even membrane protein insertion is not GTP-dependent and proceeds in the presence of non-hydrolysable GTP analogues. This has been shown by pioneering work in the Walter and Gilmore labs. GTP hydrolysis is merely required for SRP-FtsY dissociation and a GTP-dependency would only be detectable when SRP/FtsY are highly sub-stoichiometric to their substrate and would therefore need to be recycled. In the in vitro system, GTP and other nucleotides were present and this has been specified.*

21. Fig. 4D. The event is clearly post-translational. Again, is this a time-dependent, temperature-dependent reaction? This needs to be determined. From the looks of it, the apparent membrane insertion may be only a binding event that immediately leads to insertion of the small substrate. It is now simple to mechanistically dissect the contribution of the two factors but also of SecY. Given that INV work but U-INV don't, one is to assume that urea depletes both SRP and FtsY from the INVs. Although, this looks like a convincing experiment due to the differences in lanes 2,4 and 6, the "background" lane 4 is higher in previous experiments of co-translational INV association. Multiple repeats and errors are needed.

Response:

- *That urea-treatment removes SRP/FtsY from membranes is not just an assumption, but was shown in the original manuscript by immune detection of treated versus non-treated vesicles*

*(**new Fig. S5**). The fact that SRP alone does not support YohP insertion but requires the SRP receptor FtsY in addition (**new Fig. 3 & S8**), already addresses the reviewers issue. Only when FtsY is bound to SecY it can efficiently interact with SRP; this has been shown in elegant studies by the Wintermeyer group.*

- *Quantifications have been performed and the specificity of the SRP requirement was further established in proteoliposome studies (**new Fig. 6 and S8**).*

Given that this is a post-translational interaction, the authors could now address the nature of the association with the INV. For example, they can isolate the INV post mixing with the radiolabeled protein and show physical binding (and the role that SRP-FtsY play in targeting) vs insertion. If SRP acts as a receptor for such small proteins on the INV surface, it should be possible to demonstrate that (extensive) binding of YohP on the INV requires SRP.

<u>Response:</u>

- *We would like to point out that we had already shown in the original manuscript that the addition of just SRP is not sufficient for membrane insertion of YohP, but we actually need both SRP and FtsY for insertion, hence YohP insertion depends on the complete SRP pathway.*
  see also our comments above and our references to the new data that have been included.
- *We addressed this question by picking up a suggestion of reviewer 3, e.g. to check the localization of the YohP mRNA **(new Fig. 7)**. Our data show that the mRNA is already enriched at the membrane and that this occurs independent of translation.*

22. Fig. 5A. Line 278: How much SRP is used? Can more YohP be sequestered by SRP? There are no negative control proteins that should not cross-link. Lane 4 appears underloaded. How much immunoprecipitated material does it come from compared to lane 3? Compared to the input radioactivity how much is the signal in the YohP-Ffh band?

<u>Response:</u>

- In this experiment, *in vitro* synthesized YohP is used and considering that these *in vitro* systems produce client proteins at most in the low nanomolar range, the SRP concentration (100 ng/µL) is higher than the substrate concentration. Thus, we would not expect more cross-linking product by increasing the SRP concentration, in particular when we consider that the pBpa cross-linking efficiency is generally between 1 and 5%.
- It is also important to emphasize that the cross-linking product was also observed *in vivo* at the endogenous SRP concentrations (**new Fig. 5B**). Here we also included a further control by using antibodies against Trigger factor as negative control (**new Fig. 5C**).
- Lane 4 is not underloaded, but the entire material after immune-precipitation was loaded onto the gel. Since the immune-precipitation was done with α-Ffh antibodies and the radioactive signal was detected by autoradiography, only radioactive YohP that is in contact with Ffh can be visualized, explaining the almost absence of other bands.
- The amount of signal in the YohP-Ffh band is in the range of approx. 1%, as quantified by ImageJ, and thus in the range that is expected for pBpa cross-linking (e.g. Denks et al., 2017). We prefer not to include the quantification of cross-links in the manuscript, because it is a non-equilibrium method and quantification might be misleading

23. Line 281. Which protein was purified? YohP-Bpa or Ffh? If YohP-Bpa was purified, then a radioactive gel should be shown plus UV of the purified YohP-Bpa but without any immunoprecipitation, to provide some unbiased understanding of the specificity of the reaction monitored and whether YohP-Bpa interacts with other proteins.

<u>Response:</u>

- *As indicated in the original text, the Material and Methods section and also in the legend to the original Fig. 5, YohP-pBpa was enriched after in vivo cross-linking by metal affinity chromatography and the material was then separated by SDS-PAGE and decorated with α-Ffh antibodies.*

- *The sample shown in Fig. 5B is not radioactively labelled material, but reflects in vivo expressed and cross-linked YohP.*
- *Fig. 5B does not contain any immune-precipitation, but represents a western blot as indicated in material and methods and the legend.*
- *In contrast, the material in Fig. 5A is not purified and just reflects in vitro synthesized YohP that has been incubated with purified SRP and cross-linked. Fig 5A shows already the control without immune-precipitation the reviewer is asking for. In addition, we have now also tested the in vivo cross-linked material with antibodies against Trigger factor and did not see any interaction.*
- *The different experimental procedures of in vitro and in vivo cross-linking are now more clearly described.*

24. Lines 291-294. The authors offer one explanation but this is not the only one for a negative result and they are not fully exploiting the system they have set up. They can for example directly follow loss of Ffh from the INV once the insertion or the targeting reaction is complete. In the absence of any time-course kinetics here we do not know how these may correlate.

Response:

- *It would be exciting to perform fast kinetic experiments as the reviewer suggests (see also No. 15 above). However, at this stage we feel that this is beyond the scope of our study. Right now, our in vitro studies are based on radioactively labeled YohP with a very sensitive read-out, but the total concentration of in vitro synthesized YohP is in the low nanomolar/picomolar range. For real kinetic measurements, we would need defined concentrations of chemical amounts of YohP. Attempts to synthesize the YohP peptide failed due to its high hydrophobicity and therefore performing experiments with chemical amounts is not possible. Expressing YohP in vivo works as we show here, but we can maximally enrich it by chromatographic procedures; purification to almost purity failed dues to aggregation problems.*

25. Lines 332-333. For stable binding to free SRP or ribosome-bound SRP?

Response:

- *As we discuss in this section co-translational binding of SRP to substrates, it means ribosome-bound SRP*

26. Discussion statements need to cross-refer to data.

Response:

- *This has been included.*

27. Lines 378-415. While there is some interest on use of ipomF, only a very preliminary analysis was done here and the possibility of artefacts of using a 4mM compounds cannot be excluded. Detailed characterization of the compound would need a different study. I don't think this deserves the allocated space here and is over-discussed.

Response:

- *The discussion has been shortened as requested.*
