# Supplementary material for: Posttranslational insertion of small membrane proteins by the bacterial signal recognition particle
Source: PLoS Biol. 2020 Sep 30;18(9):e3000874. doi: 10.1371/journal.pbio.3000874 (PMC7549839; doi:10.1371/journal.pbio.3000874)
Supplement: Attachment — Submitted filename: Response to reveiwer.pdf [file pbio.3000874.s013.pdf]

<u>Response to Editor and Reviewers</u>

Dear Dr Koch,

Thank you very much for submitting a revised version of your manuscript "Post-translational insertion of small membrane proteins by the bacterial signal recognition particle" for consideration as a Research Article at PLOS Biology. This revised version of your manuscript has been evaluated by the PLOS Biology editors and by the original Academic Editor and reviewers 1, 2, and 4.

In light of the reviews (below), we are positive about your study and pleased to offer you the opportunity to address the lingering points raised by the reviewer. As you will see, reviewer 1 thinks that additional in vivo data could resolve standing ambiguities in your study. Together with the Academic Editor, we encourage you to seriously consider this request. Although we will not make addressing it experimentally a prerequisite for publication, adding these additional data seems relatively straightforward and would significantly strengthen the robustness and in vivo relevance of the work. Please also address the outstanding minor concerns of reviewers 1 and 2.

We cannot make any decision about publication until we have seen the revised manuscript and your response to the reviewers' comments. Your revised manuscript is also likely to be sent for further evaluation by the reviewers.

We expect to receive your revised manuscript within 2 months.

<u>Response:</u>

- We thank the Editors and Reviewers for their support in improving our manuscript.
- We have tried several *in vivo* approaches for validating further the SRP-dependency of YohP insertion. Unfortunately, we failed to observe reproducible results. It is important to emphasize that both SRP and FtsY are essential and therefore the experimental approach is to use conditional depletion strains and grow them over several generations (usually 6-8 h) under non-permissive conditions. With this approach, we can follow the depletion of SRP, but cells are barely viable and rapidly lyse, making cell fractionation studies followed by immune-detection highly unreproducible.
- We also tried pulse-labeling of YohP upon SRP-depletion, but the amounts of YohP that are synthesized in the presence or absence of SRP are much different, which precludes any reliable conclusion.
- Thus, we are sorry, but we cannot provide further in vivo evidence for the SRP-involvement in YohP insertion, with the exception of the proof of an in vivo interaction between YohP and SRP. These data are displayed in Fig. 5B. We are fully aware that conditional SRP-depletion strains have been used by other groups to determine SRP-dependency in vivo, but in our hands these experiments lack the reproducibility that is mandatory for including them in a publication.
- We have nevertheless included examples of both the cell fractionation upon SRP-depletion and pulse labeling experiments in our response to reviewer 1. Although they support the SRP-dependent insertion of YohP in vivo, we prefer to not publish them due to the low reproducibility.

  Despite these limitation, we hope that you can value our attempts to address the in vivo issue, although we did not manage to generate reliable results.

****

REVIEWS:

Reviewer #1: The authors have addressed most of my comments during the last round of review. In general, biochemical data are much stronger and provide strong evidence that YohP can be post-translationally targeted by SRP/FtsY. Additional reconstitutions using proteoliposomes further pointed to SecYEG or YidC as potential translocases for YohP, which is a nice addition. Overall the paper is much stronger. My major remaining concern is still the lack of in vivo data to show that YohP insertion is SRP/FtsY dependent and post-translational. The additional in vivo experiment showing the membrane localization of the YohP mRNA further introduces ambiguity to the interpretation of the targeting route in vivo. Since the authors already have an in vivo assay for the membrane localization of YohP by pulse chase, it seems within their reach to at least show that YohP insertion in vivo is SRP/FtsY dependent.

<u>Response:</u>

- We completely agree with the reviewer that besides the already demonstrated in vivo interaction of YohP with SRP (Fig. 5B), further in vivo data on the SRP-dependent insertion would be desirable.
- We tried two approaches: In the first approach, we performed cell fractionation studies of SRP-depleted *E. coli* cells that expressed YohP. In most experiments, SRP-depleted cells co-expressing YohP lysed before a significant reduction of the SRP levels were reached. Occasionally, we were able to harvest the cells before cell lysis and these data indeed show a drastic reduction of membrane inserted YohP (P150; Figure 1 for review), in line with an SRP-dependent insertion of YohP. Upon SRP-depletion, the vast majority of YohP was present in pellet fraction (P30) after the first centrifugation, likely representing inclusions bodies and aggregates.

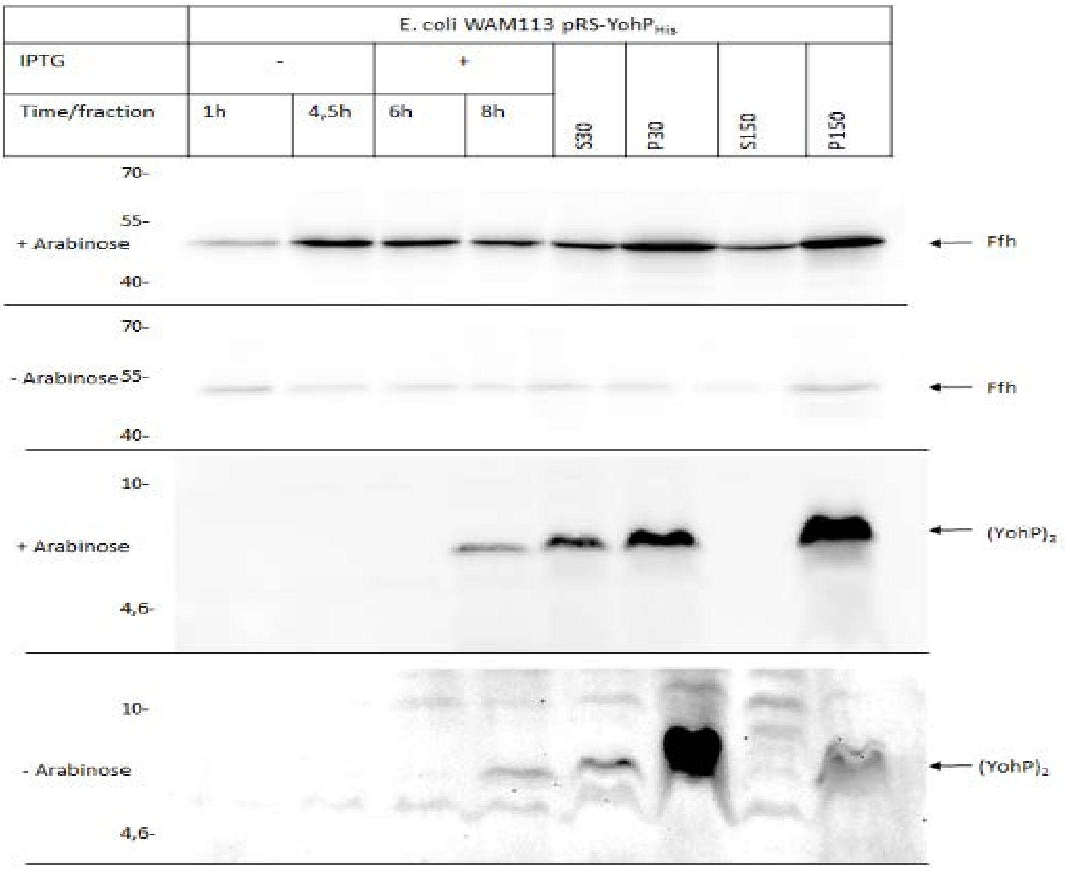

Figure 1 for review: *The conditional Ffh (SRP)-depletion strain Wam113 containing the YohP expression plasmid pRS-YohP$_{His}$, was grown in the presence (+ Ffh) or absence of arabinose (-Ffh). IPTG for induction of YohP was added after 6 hours of growth and cells were fractionated after 2 additional hours of growth. Indicated is the soluble cell extract after cell breakage and centrifugation (S30) and the corresponding pellet fraction (P30), containing unbroken cells, large aggregates and inclusion bodies. The S30 extract was then further separated by centrifugation in the cytosolic fraction (S150) and the crude membrane fraction (P150). Fractions were decorated with α-Ffh antibodies (upper two panels) or α-His antibodies (lower two panels). Please note that the lowest panel, i.e. expression of YohP upon Ffh-depletion required significantly longer exposure for detection, indicating a much lower expression or faster degradation of YohP upon SRP-depletion.*

- However, we were unable to observe these results in all repetition experiments and due to the lack of reproducibility, we don't think that these data should be included in the manuscript.

- We also attempted to isolate the inner membrane fraction (INV) of SRP-depleted cells for carbonate extraction experiments. These data show that purified INV contain much less of YohP (and the control membrane protein YfgM), in line with a reduced insertion of YohP upon SRP-depletion (Figure 2 for review). The small amounts of YohP that are detectable upon SRP depletion, are however carbonate resistant, indicating that they are membrane inserted. This further suggests that upon SRP depletion, the membrane insertion of YohP is significantly reduced and the non-inserted YohP accumulates in inclusion bodies (Figure 1 for review).

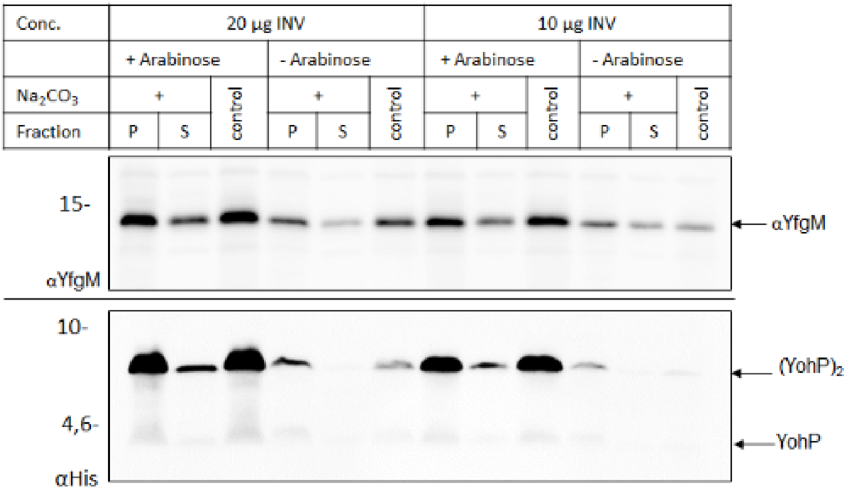

Figure 2 for review: *Inner membrane vesicles (INV) of the conditional Ffh (SRP)-depletion strain Wam113 containing the YohP expression plasmid pRS-YohP$_{His}$, were isolated under permissive conditions (+ arabinose; + Ffh) and non-permissive conditions (- arabinose, -Ffh). These INVs were then carbonate extracted to differentiate between membrane inserted material (P) and non-inserted material (S). Non-treated INV served as control. After separation by SDS-PAGE and Western blot, the levels of YohP were detected by α-His antibodies and the levels of the control protein YfgM by polyclonal antibodies against YfgM. The experiment was performed twice with different amounts of INV (10µg and 20µg total protein).*

- Although both data sets are in line with the SRP-dependent insertion of YohP, we were unable to reliable perform these experiments. This variation of *in vivo* SRP depletion experiments is probably the result of the documented and almost impossible to control metabolic re-programming upon the long term SRP depletion that is necessary to achieve a significant SRP reduction. This has been documented in several studies: https://www.jbc.org/content/286/6/4598; https://doi.org/10.1016/j.jmb.2017.12.004; https://pubmed.ncbi.nlm.nih.gov/19749044/; https://pubmed.ncbi.nlm.nih.gov/11244056/ .

- We also tried the pulse-chase approach as reviewer 1 suggested. However, the amount of pulse-labeled YohP that is produced in SRP-depleted cells is much lower than in SRP-

containing cells (Figure 3 for review). This is probably expected, considering that SRP-depleted cells are barely viable. Attempts to reduce the incubation time under non-permissive conditions were also not successful, because then significant amounts of SRP were still detectable.

Strain	WAM121 λara:ffh pRS1 YohP 6xHis											
0.2% ara	+						-					
Time(min)	2	5	10	15	30	60	2	5	10	15	30	60

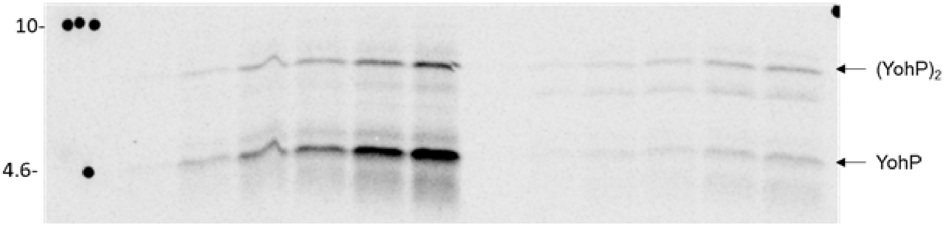

Figure 3 for review: *The conditional Ffh (SRP)-depletion strain Wam113 containing the YohP expression plasmid pRS-YohP$_{His}$, was grown under permissive conditions (+ arabinose; + Ffh) and non-permissive conditions (- arabinose, -Ffh) in LB medium and subsequently transferred to M63 minimal medium. Production of T7 RNA polymerase was induced by the addition of IPTG and after 30 min rifampicin was added to block the endogenous RNA polymerase. Cells were washed and transferred to M63 minimal medium lacking methionine and cysteine, before ^{35}S-labeled methionine/cysteine was added. Samples were withdrawn at the indicated time points, separated by SDS-PAGE and analyzed by phosphor imaging.*

Additional minor comments:

The topology of YohP via proteinase K is opposite of the conclusion in the previous draft using a different method. Given that YohP is small, could its topology be influenced by the His6 tag? I note that the authors have already shown good evidence that YohP is membrane-localized / inserted, so the topology information is not necessary in this paper and may be left for a different characterization given the current ambiguity.

Response:

- We cannot entirely exclude that the His-tag can influence the topology of YohP. However, neither our in vivo data nor our in vitro data give any indication that this is indeed the case.
- We are fully aware that in the first version of the manuscript we had proposed a different YohP topology based on AMS labeling. We later also noticed that AMS labeling gives different results in vivo and in vitro, while the proteinase K based approach that is shown in the current manuscript, gives consistent results both in vivo and in vitro.

Since OmpA translocation is SecA and SecYEG-dependent, is it possible that the effect of IpomF on OmpA translocation could be due to inhibition of SecA, rather than SecYEG?

Response:

- This is a valid point and we cannot entirely exclude that IpomF also acts on SecA. However, as YohP insertion is SecA independent and still inhibited by IpomF, we think that the primary target of IpomF is the SecYEG complex.

Reviewer #2: The authors have made a commendable effort to address the reviewers' comments.

1. However, many of their statements are still too definitive (for example in the abstract). The authors should simply state their observations for the constructs they examined and the assays they carried out. As just a few examples, I suggest the following changes (these are just examples, modifications need to be made throughout the manuscript):
--line 194: "…these data show that the vast majority of YohP-His is oriented in a Cin-Nout topology, …"
--line 223: "…does not efficiently occur spontaneously in our assay, suggesting that insertion requires…"
--line 267: "…strongly stimulated YohP-His insertion."
--lines 298-299: "In order to further test whether…"
--line 366: "…whereas our data indicate that the…"
--line 370: "To obtain further information about the insertion mode…"
Small proteins are tricky to study and as found by the authors, different assays can lead to completely different conclusions. In the previous draft, the authors concluded, based on AMS labeling, that YohP has a Nin-Cout topology. Now the authors conclude the protein has a Cin-Nout topology. Both assays have limitations. The authors should avoid stating that they have "proof".

2. Some non-standard phrasing that should be changed:
--line 203: "allows to determine"
--line 233: "for preventing possible saturation"
--line 283: "10-fold up-scaled"
--line 326: "were UV-exposed"
--line 329: "decorated with a-Ffh antibodies"

Response:

- We thank the reviewer for the support. In light of the reviewer's comments, we have modified our statements as suggested by the reviewer. In addition, we went through the entire manuscript and have modified further statements that could be perceived as too definitive. All changes are highlighted (track-change) in a text file that is submitted along with a main manuscript.

- We have also changed the non-standard phrasing as requested.

Reviewer #4: I find that the authors have done a formidable job in addressing the reviewers comments and now present a much stronger case for they study with more controls and clarification of issues that were confusing or not directly justified by the available data.
I therefore fully support its publication.

Response:

- We thank the reviewer for the support.
