# Supplementary material for: Posttranslational insertion of small membrane proteins by the bacterial signal recognition particle
Source: PLoS Biol. 2020 Sep 30;18(9):e3000874. doi: 10.1371/journal.pbio.3000874 (PMC7549839; doi:10.1371/journal.pbio.3000874)
Supplement: Attachment — Submitted filename: Response to reviewer.docx [file pbio.3000874.s014.docx]

**Response to Editor and Reviewers**

DATA AVAILABILITY:

Please rename your "Quantification" file as S1 Data, making sure it has a legend and is mentioned in each figure legend.

**Response:**

This was changed
